# Correction: Climate, Demography, and Zoogeography Predict Introgression Thresholds in Salmonid Hybrid Zones in Rocky Mountain Streams

**DOI:** 10.1371/journal.pone.0167711

**Published:** 2016-12-01

**Authors:** The PLOS ONE Staff

## Notice of Republication

This article was republished on November 18, 2016 to correct errors in the author byline. The publisher apologizes for the errors. Please download this article again to view the correct version. The originally published, uncorrected article and the republished, corrected articles are provided here for reference.

## Supporting Information

S1 File Originally published, uncorrected article. (PDF)

S2 File Republished corrected article. (PDF)
