# Supplementary material for: Climate, Demography, and Zoogeography Predict Introgression Thresholds in Salmonid Hybrid Zones in Rocky Mountain Streams
Source: PLoS One. 2016 Nov 9;11(11):e0163563. doi: 10.1371/journal.pone.0163563 (PMC5102351; doi:10.1371/journal.pone.0163563)
Supplement: S1 File — Tables A–E. Table A. Studies used in the synthetic analysis. Data from studies included in the synthetic analysis of introgression between westslope cutthroat trout and rainbow trout (RT). These include the published source, number of sample sites inside and outside the historical range of rainbow trout, number of genotyped fish, marker type, and the means (ranges) for each dependent variable. Table B. Candidate variables. Candidate variables considered in the logistic models, the rationale for their inclusion and expected effect, and supporting citations. Table C. Covariate correlation matrix. Correlations among environmental covariates and introgression estimates at the 558 stream sites in the dataset. Table D. Model selection results for 10% PRTA. Model selection results for logistic regression equations relating environmental covariates to whether sites exceeded 10% rainbow trout alleles (PRTA). The 20 top models are ranked from most to least plausible. Underlined variables had coefficients that were not significantly different from zero. The top-ranked model is the consensus model. Table E. Study region statistics. Descriptive statistics for model covariates throughout the 55,234-km stream network in the study area. (DOCX) [file pone.0163563.s001.docx]

**Table A. Studies used in the synthetic analysis.**

|  | Sites | |  |  | Dependent variables^c^ | |
| --- | --- | --- | --- | --- | --- | --- |
| Source*^a^* | RT introduced | RT native | Fish | Markers^b^ | PRTA | PFRT |
| [1] | 27 | 0 | 751 | M | 13.3 (0–91.6) | 0(0)^d^ |
| [2] | 0 | 3 | 143 | M | 78.8 (54.3–96.8) | 79.5 (56.3–96.8) |
| [3] | 22 | 10 | 751 | M | 4.7 (0–76.0) | 10.6 (0–97.4) |
| [4] | 25 | 0 | 248 | M | 52.2 (0–100) | 56.9 (0–100) |
| [5] | 50 | 0 | 1188 | M | 22.1 (0–90.0) | 40.3 (0–100) |
| [6] | 56 | 0 | 1462 | M | 9.3 (0–98.0) | 19.4 (0–100) |
| [7] | 0 | 17 | 839 | M | 35.4 (0–90.1) | 40.9 (96.5) |
| [8] | 6 | 0 | 232 | A | 18.8 (0–85.3) | 0(0)^d^ |
| [9] | 0 | 32 | 1274 | M | 7.5 (0–30.3) | 0(0)^d^ |
| [10] | 138 | 41 | 3737 | S | 11.2 (0–99.6) | 24.9 (0–100) |
| [11] | 6 | 33 | 850 | M | 48.9 (0–100) | 59.1 (0–100) |
| [12] | 0 | 92 | 1840^e^ | P | 0(0)^d^ | 33.2 (0–100) |

Data from studies included in the meta-analysis of introgression between westslope cutthroat trout and rainbow trout (RT). These include the published source, number of sample sites inside and outside the historical range of rainbow trout, number of genotyped fish, marker type, and the means (ranges) for each dependent variable.

*^a^*1. Boyer MC, Muhlfeld CC, Allendorf FW. Rainbow trout (*Oncorhynchus mykiss*) invasion and the spread of hybridization with native westslope cutthroat trout (*Oncorhynchus clarkii lewisi*). Can J Fish Aquat Sci. 2008; 65: 658–669.

2. Campbell M, Cegelski C. Native species investigations. Grant # F-73-R-25, July 1, 2002 to June 30, 2003. Idaho Department of Fish and Game Report 03-49. 2003. 46 p.

3. Campbell M, Kozfkay C. Native species investigations. Grant # F-73-R-25, July 1, 2005 to June 30, 2006. Idaho Department of Fish and Game Report 08-01. 2008. 37 p.

4. Campbell MR, Ryan R, Heindel K. Origin and extent of rainbow trout hybridization and introgression of westslope cutthroat trout populations in the Pend Oreille drainage, Idaho. Idaho Department of Fish and Game Report 13-16. 2013. 25 p.

5. Carim K, Eby L, Pierce R. Does whirling disease mediate hybridization between a native and nonnative trout? N Am J Fish Manag. 2015; 35: 337–351.

6. Corsi MP. Management and life history consequences of hybridization between westslope cutthroat trout (*Oncorhynchus clarkii lewisi*) and rainbow trout (*Oncorhynchus mykiss*). Ph.D. dissertation, University of Montana, Missoula. 2011.

7. Kozfkay CC, Campbell MR, Yundt SP, Peterson MP, Powell MS. Incidence of hybridization between naturally sympatric westslope cutthroat trout and rainbow trout in the Middle Fork Salmon River drainage, Idaho. Trans Am Fish Soc. 2007; 136: 624–638.

8. Leary RF, Allendorf FW, Phelps SR, Knudsen KL. Introgression between westslope cutthroat and rainbow trout in the Clark Fork River drainage. Proc Montana Acad Sci. 1984; 43: 1–18.

9. Loxterman JL, Keeley ER, Njoroge ZM. Evaluating the influence of stocking history and barriers to movement on the spatial extent of hybridization between westslope cutthroat trout and rainbow trout. Can J Fish Aquat Sci. 2014; 71: 1050–1058.

10. McKelvey KS, Young MK, Wilcox TM, Bingham DM, Pilgrim KL, Schwartz MK. Patterns of hybridization among cutthroat trout and rainbow trout in northern Rocky Mountain streams. Ecol Evol. 2016; 6: 688–706.

11. Paragamian VL, Walters J, Maiolie M, Handley K, Campbell M, Kozfkay C, et al. Kootenai River fisheries investigations: salmonid studies. Annual progress report May 1, 2007–April 30, 2008. Idaho Department of Fish and Game Report 08-21. 2008. 52 p.

12. Weigel DE, Peterson JT, Spruell P. Introgressive hybridization between native cutthroat trout and introduced rainbow trout. Ecol Appl. 2003; 13: 38–50.

^b^A, allozymes; M, microsatellites or other co-dominant markers; P, paired interspersed nuclear elements; S, single nucleotide polymorphisms.

^c^PRTA, percentage of rainbow trout alleles at a site; PFRT, percentage of fish with rainbow trout alleles at a site.

^d^Authors did not report this variable, but for sites where PRTA or PFRT was zero, the other variable was set to zero.

^e^Estimated based on sample sizes in [12].

**Table B. Candidate variables.**

| Variable | Rationale | Citations^a^ |
| --- | --- | --- |
| *Abiotic* | | |
| T: mean August temperature (°C) | Declining temperature (or its surrogate, increasing elevation) is related to decreases in rainbow trout presence and introgression. Rainbow trout have metabolic rates, growth efficiencies, oxygen consumption rates, and life histories that are better adapted to warmer, more productive habitats. | [7,11,17,18,21,23,34,40,61,86,94,95,101–105] |
| S: slope (%) | Increasing slope may lead to greater bioenergetic costs for upstream migrating rainbow trout. Many salmonid species show reductions in habitat occupancy with greater slope. | [23,40,59,101,106–108] |
| MAF: mean annual flow (m^3^/s) | Larger, more productive streams are associated with rainbow trout, and smaller, less productive streams are associated with cutthroat trout. | [23,61,96,101,104,106,107,109,110] |
| CFM: center of flow mass, the date when 50% of the mean annual flow has been discharged | High snowmelt-driven flows in late spring and early summer are associated with declines in rainbow trout recruitment. | [85,111–114] |
| W95: number of winter days with flows among the top 5% for the year | High winter flows are positively related to rainbow trout presence and negatively related to cutthroat trout presence. | [23,59] |
| E, N: easting and northing (m) | Geographic location can serve as a surrogate for climatic and geological covariates not otherwise represented. | [51] |
| *Biotic* | | |
| DT13: Distance (m) to mean August temperature > 13 °C | Warmer streams may favor rainbow trout. Occurrence of rainbow trout peaked at this temperature in this region. | [6,7,8,11,29,35,36,95,115–117] |
| DF3: Distance (m) to mean annual flow > 2.83 m^3^/s | Larger streams may favor rainbow trout. This threshold exceeds those habitats generally suitable for cutthroat trout spawning. | See previous |
| DS: Shortest distance (m) to rainbow trout | The shortest distance among: 1) the two previous variables, 2) habitat known to support a naturally reproducing population of rainbow trout, or 3) habitat stocked with rainbow trout within 10 years of the time of genetic sampling. Proximity to any of these four habitats is a surrogate for proximity to rainbow trout propagules. | See previous |
| RTrange: historical range of rainbow trout (yes/no) | Occupancy over evolutionary time enabled rainbow trout to colonize a larger portion of a watershed, for hybrid zones to stabilize at their highest longitudinal point, and for levels of introgression to achieve a quasi-equilibrium. In some cases, being in the range of rainbow trout equates to exposure to the more fecund, anadromous form, steelhead, which may increase propagule pressure from rainbow trout. | [18,74,80] |
| YCTI: Yellowstone cutthroat trout introgression (yes/no) | Populations of westslope cutthroat trout may form hybrid swarms after contact with Yellowstone cutthroat trout. Three-way hybrids with rainbow trout can be common in these hybrid swarms, suggesting that hybridization between the cutthroat trout subspecies may form a genetic bridge to introgression with rainbow trout. | [18,118–121] |

Candidate variables considered in the logistic models, the rationale for their inclusion and expected effect, and supporting citations.

^a^Reference numbering continues from the online text.

101. Bozek MA, Hubert WA. Segregation of resident trout in streams as predicted by three habitat dimensions. Can J Zool. 1992; 70: 886-890.

102. Paul AJ, Post JR. Spatial distribution of native and nonnative salmonids in streams of the eastern slopes of the Canadian Rocky Mountains. Trans Am Fish Soc. 2001; 130: 417–430.

103. Ostberg CO, Rodriguez RJ. Hybridization and cytonuclear associations among native westslope cutthroat trout, introduced rainbow trout, and their hybrids within the Stehekin River drainage, North Cascades National Park. Trans Am Fish Soc. 2006; 135: 924–942.

104. Rasmussen JB, Robinson MD, Heath DD. Ecological consequences of hybridization between native westslope cutthroat (*Oncorhynchus clarkii lewisi*) and introduced rainbow (*Oncorhynchus mykiss*) trout: effects on life history and habitat use. Can J Fish Aquat Sci. 2010; 67: 357–370

105. Yau MM, Taylor EB. Cold tolerance performance of westslope cutthroat trout (*Oncorhynchus clarkii lewisi*) and rainbow trout (*Oncorhynchus mykiss*) and its potential role in influencing interspecific hybridization. Can J Zool. 2014; 92: 777–784.

106. Hartman GF, Gill CA. Distributions of juvenile steelhead and cutthroat trout (*Salmo gairdneri* and *S. clarki clarki*) within streams in southwestern British Columbia. J Fish Res Board Can. 1968; 25: 33–48.

107. Campton DE, Utter FM. Natural hybridization between steelhead trout (*Salmo gairdneri*) and coastal cutthroat trout (*S. clarki*) in two Puget Sound streams. Can J Fish Aquat Sci. 1985; 42: 110–119.

108. Fausch KD. Do gradient and temperature affect distributions of, and interactions between, brook charr (*Salvelinus fontinalis*) and other resident salmonids in streams? Physiol Ecol Japan. 1989; 1: 303–322.

109. Platts WS. Relationships among stream order, fish populations, and aquatic geomorphology in an Idaho river drainage. Fisheries. 1979; 4(2): 5–9.

110. Rosenfeld J, Porter M, Parkinson E. Habitat factors affecting the abundance and distribution of juvenile cutthroat trout (*Oncorhynchus clarki*) and coho salmon (*Oncorhynchus kisutch*). Can J Fish Aquat Sci. 2000; 57: 766–774.

111. Seegrist DW, Gard R. Effects of floods on trout in Sagehen Creek, California. Trans Am Fish Soc. 1972; 102: 478–482.

112. Nehring RB, Anderson RM. Determination of population-limiting critical salmonid habitats in Colorado streams using the Physical Habitat Simulation system. Rivers. 1993; 4: 1–19.

113. Latterell JJ, Fausch KD, Gowan C, Riley SC. Relationship of trout recruitment to snowmelt runoff flows and adult trout abundance in six Colorado mountain streams. Rivers. 1998; 6: 240–250.

114. Fausch KD, Taniguchi Y, Nakano S, Grossman GD, Townsend CR. Flood disturbance regimes influence rainbow trout invasion success among five holarctic regions. Ecol Appl. 2001; 11: 1438–1455.

115. Bennett SN, Kershner JL. Levels of introgression in westslope cutthroat trout populations nine years after changes to rainbow trout stocking programs in southeastern British Columbia. N Am J Fish Manag. 2009; 29: 1271–1282.

116. Kovach RP, Eby LA, Corsi MP. Hybridization between Yellowstone cutthroat trout and rainbow trout in the upper Snake River basin, Wyoming. N Am J Fish Manag. 2011; 31: 1077–1087.

117. Marie AD, Bernatchez L, Garant D. Environmental factors correlate with hybridization in stocked brook charr (*Salvelinus fontinalis*). Can J Fish Aquat Sci. 2012; 69: 884–893.

118. Marnell LF, Behnke RJ, Allendorf FW. Genetic identification of cutthroat trout, *Salmo clarki*, in Glacier National Park, Montana. Can J Fish Aquat Sci. 1987; 44: 1830–1839.

119. Forbes SH, Allendorf FW. Associations between mitochondrial and nuclear genotypes in cutthroat hybrid swarms. Evolution. 1991; 45: 1332–1349.

120. Mandeville EG, Parchman TL, McDonald DB, Buerkle CA. Highly variable reproductive isolation among pairs of *Catostomus* species. Mol Ecol. 2015; 24: 1856–1872.

121. Pritchard VL, Garza JC, Peacock MM. SNPs reveal previously undocumented non-native introgression within threatened trout populations. Conserv Genet. 2015; 16: 1001–1006.

**Table C. Covariate correlation matrix.**

|  | T | S | CFM | W95 | MAF | DF3 | DT13 | DS | N | E | PRTA |
| --- | --- | --- | --- | --- | --- | --- | --- | --- | --- | --- | --- |
| S | -0.27 |  |  |  |  |  |  |  |  |  |  |
| CFM | -0.32 | 0.00 |  |  |  |  |  |  |  |  |  |
| W95 | 0.33 | -0.03 | -0.84 |  |  |  |  |  |  |  |  |
| MAF | 0.34 | -0.40 | 0.20 | -0.23 |  |  |  |  |  |  |  |
| DF3 | -0.33 | -0.05 | 0.08 | -0.07 | -0.29 |  |  |  |  |  |  |
| DT13 | -0.60 | -0.03 | 0.33 | -0.30 | -0.04 | 0.27 |  |  |  |  |  |
| DS | -0.60 | -0.01 | 0.24 | -0.20 | -0.28 | 0.65 | 0.55 |  |  |  |  |
| N | 0.12 | 0.04 | -0.13 | -0.03 | 0.10 | -0.13 | -0.10 | -0.20 |  |  |  |
| E | -0.29 | -0.19 | 0.09 | 0.06 | -0.14 | 0.20 | 0.27 | 0.25 | -0.38 |  |  |
| PRTA | 0.50 | -0.18 | -0.10 | 0.05 | 0.29 | -0.20 | -0.34 | -0.35 | 0.11 | -0.13 |  |
| PFRT | 0.55 | -0.24 | -0.12 | 0.13 | 0.33 | -0.24 | -0.33 | -0.42 | 0.03 | -0.08 | 0.89 |

Correlations among environmental covariates and introgression estimates at the 558 stream sites in the dataset.

**Table D. Model selection results for 10% PRTA.**

| Rank | Model | AIC |
| --- | --- | --- |
| 1 | T + RTrange + DS + DT13 + MAF + E | 433.13 |
| 2 | T + RTrange + DS + DT13 + MAF + E + YCTI | 433.51 |
| 3 | T + RTrange + DS + DT13 + MAF + E + W95 | 434.27 |
| 4 | T + RTrange + DS + DT13 + MAF + E + S | 434.32 |
| 5 | T + RTrange + DS + DT13 + MAF + E + S + YCTI | 434.61 |
| 6 | T + RTrange + DS + DT13 + MAF + E + W95 + YCTI | 435.06 |
| 7 | T + RTrange + DS + DT13 + MAF + E + DF3 | 435.11 |
| 8 | T + RTrange + DS + DT13 + MAF + E + S + W95 | 435.28 |
| 9 | T + RTrange + DS + DT13 + MAF + E + DF3 + YCTI | 435.34 |
| 10 | T + RTrange + DS + DT13 + MAF + E + S + W95 + YCTI | 436.02 |
| 11 | T + RTrange + DS + DT13 + MAF + E + DF3 + W95 | 436.21 |
| 12 | T + RTrange + DS + DT13 + MAF + E + DF3 + S | 436.28 |
| 13 | T + RTrange + DS + DT13 + MAF + E + DF3 + S + YCTI | 436.38 |
| 14 | T + RTrange + DS + DT13 + E + S + W95 | 436.54 |
| 15 | T + RTrange + DS + DT13 + MAF + E + DF3 + W95 + YCTI | 436.85 |
| 16 | RTrange + DS + DT13 + MAF + E + S | 436.97 |
| 17 | RTrange + DS + DT13 + MAF + E + S + YCTI | 437.08 |
| 18 | T + RTrange + DS + DT13 + MAF + E + DF3 + S + W95 | 437.18 |
| 19 | T + RTrange + DS + DT13 + E + S + W95 + YCTI | 437.61 |
| 20 | T + RTrange + DS + DT13 + MAF + E + DF3 + S + W95 + YCTI | 437.73 |
|  | T | 484.96 |
|  | DS | 495.27 |
|  | DT13 | 499.36 |
|  | MAF | 557.78 |
|  | RTrange | 579.24 |
|  | E | 589.13 |

Model selection results for logistic regression equations relating environmental covariates to whether sites exceeded 10% rainbow trout alleles (PRTA). The 20 top models are ranked from most to least plausible, along with single-variable models for each of the covariates in the consensus model. Underlined variables had coefficients that were not significantly different from zero. The top-ranked model is the consensus model.

**Table E. Descriptive covariate statistics.**

| Variable | Mean | Median | SD | Minimum | Maximum |
| --- | --- | --- | --- | --- | --- |
| T (°C) | 11.73 | 11.50 | 2.67 | 3.91 | 27.36 |
| S (%) | 0.037 | 0.030 | 0.029 | 0 | 0.100 |
| CFM (day) | 193 | 212 | 64 | 0 | 280 |
| W95 (days) | 1.24 | 0.40 | 1.79 | 0 | 18.45 |
| MAF (m^3^/s) | 0.641 | 0.221 | 1.016 | 0.028 | 5.653 |
| DF3 (m) | 19374 | 11559 | 31126 | 0 | 200000 |
| DT13 (m) | 13211 | 5898 | 24690 | 0 | 200000 |
| DS (m) | 5785 | 2876 | 13667 | 0 | 200000 |
| N (m) | 1841862 | 1851978 | 140886 | 1516682 | 2098463 |
| E (m) | 1535116 | 1539858 | 142958 | 1234229 | 1862914 |

Descriptive statistics for model covariates throughout the 55,234-km stream network in the study area.
