# Supplementary material for: Correction: Climate, Demography, and Zoogeography Predict Introgression Thresholds in Salmonid Hybrid Zones in Rocky Mountain Streams
Source: PLoS One. 2016 Dec 1;11(12):e0167711. doi: 10.1371/journal.pone.0167711 (PMC5131941; doi:10.1371/journal.pone.0167711)
Supplement: S1 File — (PDF) [file pone.0167711.s001.PDF]

RESEARCH ARTICLE

Climate, Demography, and Zoogeography Predict Introgression Thresholds in Salmonid Hybrid Zones in Rocky Mountain Streams

Michael K. Young[1]*, Daniel J. Isaak[2], Kevin S. McKelvey[1], Taylor M. Wilcox[1,3], Daniel M. Bingham[1¤], Kristine L. Pilgrim[1], Kellie J. Carim[1], Matthew R. Campbell[4], Matthew P. Corsi[5], Dona L. Horan[2], David E. Nagel[2], Michael K. Schwartz[1]

1 National Genomics Center for Wildlife and Fish Conservation, Rocky Mountain Research Station, USDA Forest Service, Missoula, Montana, United States of America, **2** Rocky Mountain Research Station, USDA Forest Service, Boise, Idaho, United States of America, **3** Division of Biological Sciences, University of Montana, Missoula, Montana, United States of America, **4** Eagle Fish Genetics Laboratory, Idaho Department of Fish and Game, Eagle, Idaho, United States of America, **5** Idaho Department of Fish and Game, Coeur d'Alene, Idaho, United States of America

¤ Current address: Rogue Biological Consultants, Vancouver, Washington, United States of America
* mkyoung@fs.fed.us

OPEN ACCESS

Citation: Young MK, Isaak DJ, McKelvey KS, Wilcox TM, Bingham DM, Pilgrim KL, et al. (2016) Climate, Demography, and Zoogeography Predict Introgression Thresholds in Salmonid Hybrid Zones in Rocky Mountain Streams. PLoS ONE 11 (11): e0163563. doi:10.1371/journal.pone.0163563

Editor: Peter Prentis, Queensland University of Technology, AUSTRALIA

Received: July 1, 2016

Accepted: September 11, 2016

Published: November 9, 2016

Copyright: This is an open access article, free of all copyright, and may be freely reproduced, distributed, transmitted, modified, built upon, or otherwise used by anyone for any lawful purpose. The work is made available under the Creative Commons CC0 public domain dedication.

Data Availability Statement: Data have been deposited to Dryad: doi:10.5061/dryad.73s0n.

Funding: Funding for this work was provided by Region 1 of the U.S. Forest Service. The funder provided support in the form of salaries for authors [DMB], but did not have any additional role in the study design, data collection and analysis, decision to publish, or preparation of the manuscript. The specific roles of these authors are articulated in the 'author contributions' section.

Abstract

Among the many threats posed by invasions of nonnative species is introgressive hybridization, which can lead to the genomic extinction of native taxa. This phenomenon is regarded as common and perhaps inevitable among native cutthroat trout and introduced rainbow trout in western North America, despite that these taxa naturally co-occur in some locations. We conducted a synthetic analysis of 13,315 genotyped fish from 558 sites by building logistic regression models using data from geospatial stream databases and from 12 published studies of hybridization to assess whether environmental covariates could explain levels of introgression between westslope cutthroat trout and rainbow trout in the U. S. northern Rocky Mountains. A consensus model performed well (AUC, 0.78–0.86; classification success, 72–82%; 10-fold cross validation, 70–82%) and predicted that rainbow trout introgression was significantly associated with warmer water temperatures, larger streams, proximity to warmer habitats and to recent sources of rainbow trout propagules, presence within the historical range of rainbow trout, and locations further east. Assuming that water temperatures will continue to rise in response to climate change and that levels of introgression outside the historical range of rainbow trout will equilibrate with those inside that range, we applied six scenarios across a 55,234-km stream network that forecast 9.5–74.7% declines in the amount of habitat occupied by westslope cutthroat trout populations of conservation value, but not the wholesale loss of such populations. We conclude that introgression between these taxa is predictably related to environmental conditions, many of which can be manipulated to foster largely genetically intact populations of westslope cutthroat trout and help managers prioritize conservation activities.

Competing Interests: The funder provided support in the form of salaries for authors [DMB]. This does not alter our adherence to PLOS ONE policies on sharing data and materials.

Introduction

The introduction, establishment, and spread of nonnative species is considered one of the fundamental threats to the conservation of biodiversity. Following such introductions, many native species have suffered declines in abundance, contracted distributions, or even extinctions [1]. Although the latter is usually described in demographic terms, conservation geneticists consider some taxa to be at risk of genomic extinction i.e., the invasion of the genome from introgressive hybridization with a nonnative species to the extent that all individuals are hybrids and collectively constitute a hybrid swarm [2]. Perhaps the most cited example of this phenomenon involves cutthroat trout *Oncorhynchus clarkii* and rainbow trout *O. mykiss*, sister taxa that are spring-spawning salmonid fishes native to montane streams in western North America [3]. Until widespread fish stocking began in the late 19th century, rainbow trout were absent from many inland basins in which cutthroat trout were native [4]. Even though these taxa may have diverged over 10 million years ago [5], hybrids between them were regularly detected following these introductions. The ubiquity of hybrid individuals at some locations, the presence of late-generation crosses involving hybrids, and the spread of introgression away from rainbow trout introduction sites [6,7] have often led to the conclusion that anthropogenically-driven secondary contact will result in the formation of hybrid swarms and the genomic extinction of cutthroat trout populations [8,9]. Based on the logic that all matings involving hybrids will beget hybrids [10], it has been argued that even with selection against introgressed individuals ([11] but see [12,13]), indigenous populations of cutthroat trout will be unable to resist invasion of their genome by rainbow trout alleles [14].

Genetic interactions between rainbow trout and cutthroat trout are, however, more complicated. Although human-assisted sympatry between rainbow trout and cutthroat trout has been underway for ~150 years in some areas, elsewhere populations of some subspecies of cutthroat trout have co-occurred with rainbow trout for at least 20,000 years following the retreat of continental and mountain glaciers and cessation of glacial lake floods [4]. In both circumstances, genetically intact populations of cutthroat trout remain common in headwater streams [15,16]. Moreover, many populations purportedly representing hybrid swarms contain substantial numbers of nonadmixed individuals of either or both parental species and often feature a nonrandom distribution of alleles among individuals [17,18]. Both conditions imply resistance to runaway introgression, and hint at the potential for ecological segregation between parental taxa to mediate the degree of hybridization and the location of hybrid zones [19–21].

There is a growing appreciation that environmental characteristics can shape population genetic structure, including hybridization [22]. For aquatic species, main-stem to headwater gradients in habitat structure, flow regimes, productivity, and temperature are likely to influence freshwater species distributions [23] and thus hybrid zone position and characteristics [20,24,25]. Also contributing to hybridization is propagule pressure [26], which reflects the frequency and amount of dispersal of either parental form from source populations and is dependent on the strength and proximity of those populations. This mechanism may be particularly relevant for salmonid fishes because many populations are subsidized or wholly maintained at artificially high levels to provide sport fisheries [27]. Because fish stocking has been common in high-elevation lakes [28] and low-elevation streams and rivers [29], propagule pressure may arise from more than one location within a riverscape. An additional nuance is that the location of and patterns of introgression within hybrid zones may reflect the period of contact between parental species and take many generations to stabilize at some quasi-equilibrium [30], especially if secondary contact between taxa relies on changes in stream access that vary at decadal or century-long time scales. Thus, rather than interactions with rainbow trout leading to the wholesale genomic extinction of cutthroat trout, these taxa may exhibit

geographically complex and dynamic patterns of admixture that include some locations with populations of cutthroat trout that remain free from introgression. In addition, given that temperature is often a master variable dictating the distribution and abundance of aquatic ectotherms [31], climate-change-induced stream warming [32] might be expected to mobilize hybrid zones [33].

Although many studies have recognized spatial patterns in hybridization between rainbow trout and cutthroat trout [34–36], these have been limited in geographic extent or have used imprecise proxies for instream conditions (e.g., elevation or precipitation to represent stream temperature or flow) because of the absence of more accurate or direct measures. Recent development of massive geospatial stream databases provides a means to represent important environmental variables accurately and consistently across broad geographic areas [37,38]. Here, we apply these new geospatial data in a synthetic analysis (sensu [39]) of hybridization studies to examine patterns of introgression between westslope cutthroat trout *O. c. lewisi* and rainbow trout in the U.S. northern Rocky Mountains. Models are developed to describe how abiotic and biotic covariates are related to exceeding three levels of introgression (1%, 10%, and 20% rainbow trout alleles) relevant to conservation priorities for cutthroat trout. Models encompass samples from river basins where rainbow trout are native (the Salmon and Clearwater River basins in Idaho and the Kootenai River basin in northern Idaho and northwestern Montana) and where they are introduced (most of Montana and portions of northern Idaho). The models are then used to extend predictions to all streams in the study area and to estimate how future climate change and long-term exposure to rainbow trout could affect the distribution and prevalence of introgression by rainbow trout alleles in populations of cutthroat trout.

Materials and Methods

The study area encompassed 246,231 km^2 throughout the historical range of westslope cutthroat trout in Idaho and Montana [15] (Fig 1). This mountainous area is drained by a diverse network of rivers and streams that flow through mid-elevation steppe grasslands, high-elevation forests, and alpine tundra. Within this region, a network was delineated from the 1:100,000-scale National Hydrography Dataset Plus (NHDPlus) Version 2 geospatial layer (www.horizon-systems.com/NHDPlus/index.php). Reaches in the NHDPlus dataset that were coded as intermittent (Fcode = 46003) or had summer flows < 0.028 m^3/s were removed from the network to exclude areas of marginal importance to fish. We also trimmed reaches from the upper extent of the network where slopes consistently exceeded 10% because geological barriers are common in these areas and fish occurrence is rare [40], as well as reaches with mean annual flow > 5.66 m^3/s because large streams are unlikely to support spawning habitat for cutthroat trout [41,42] and thus do not constitute a part of a hybrid zone sensu stricto [43]. Application of the slope and flow criteria resulted in a 55,234-km stream network used for subsequent analyses.

Within this area, we used a spatially extensive hybridization dataset from across northern Idaho and Montana and supplemented it with local datasets for additional basins in both states (Table A in S1 File). These studies adopted an array of spatial sampling designs, from single sites in a few basins to longitudinal sampling throughout a basin or its tributaries, and combinations thereof. For data to be included in the present analyses, geographic coordinates of sample sites had to be available in publications, in online sources, or from the authors so that samples could be linked to the stream network and attributed with model covariates. These sources also had to provide one of two dependent variables for each sample site: the percentage of rainbow trout alleles among all fish (PRTA) or the percentage of fish with rainbow trout alleles (PFRT). Because a consistent stream network data layer and associated covariates were

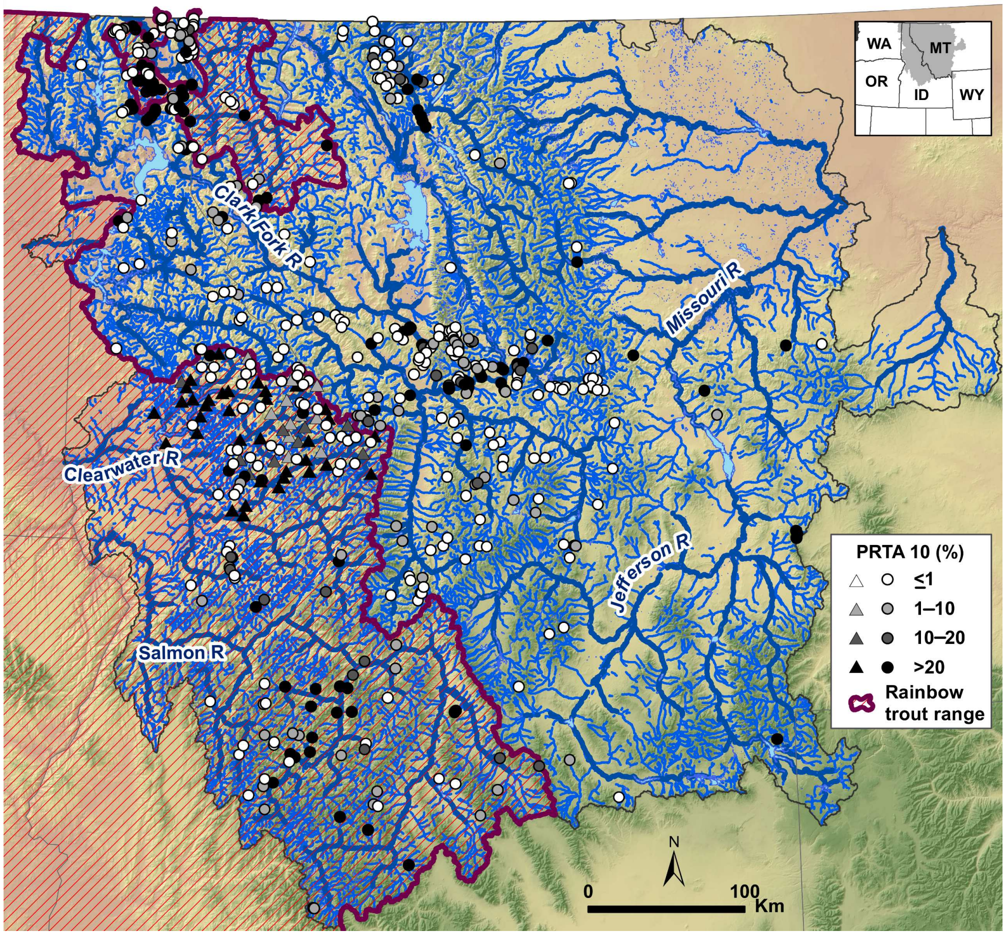

Fig 1. Sites in the synthetic analysis. Study area showing elevation hillshade, stream network, overlap in the historical ranges of rainbow trout and westslope cutthroat trout (purple hashmarks overlaying the stream network), and locations of 558 sample sites, scaled by levels of introgression (<1%, white; 1–10%, light gray; 10–20%, dark gray; >20%, black), used to develop logistic regression models. Most sites ($n = 501$) provided estimates of the percentage of rainbow trout alleles at a site (PRTA; circles). Some sites ($n = 57$) provided only estimates of the percentage of fish with rainbow trout alleles (PFRT; triangles). Thick stream lines show rivers with mean annual flows that exceeded 2.83 m^3/s.

doi:10.1371/journal.pone.0163563.g001

available only for U.S. streams, data on westslope cutthroat trout in Canada were not considered. We also constrained our analyses to studies involving westslope cutthroat trout in Idaho and Montana because the geographic coverage in other portions of its U.S. range (i.e., eastern Washington and Oregon) and for other subspecies was insufficient to characterize relations among abiotic variables and introgression. If multiple studies were based on the same data, only data in the original study were used. Sites known to be inaccessible to rainbow trout or westslope cutthroat trout because of the presence of migration barriers were not considered, but some sites included in the analysis may have had unrecognized migration barriers that would have precluded introgression. The final dataset was derived from 12 studies in which 558 sites were sampled—501 with estimates of PRTA and 512 with estimates of PFRT—and 13,315 fish were genotyped (Table A in S1 File). These data encompassed a diversity of stream environments, but those environments were broadly similar when compared across samples drawn from the native and introduced ranges of rainbow trout (Table 1). In contrast, average levels of introgression per site were higher where rainbow trout were native than where they

Table 1. Study site statistics.

Covariate[a]	RTrange	Mean	Median	SD	Minimum	Maximum
T (˚C)	No	10.7	10.7	1.9	6.0	17.5
	Yes	11.3	11.1	1.9	7.2	16.9
S (%)	No	5.0	4.3	3.61	0.1	18.9
	Yes	5.6	4.7	3.8	0.1	23.5
CFM (day)	No	197	195	13.3	166	234
	Yes	201	200	15	168	245
W95 (days)	No	3.1	2.2	2.7	0	11.1
	Yes	2.2	1.1	2.8	0	12.6
MAF (m^3/s)	No	0.55	0.27	0.69	0.002	4.43
	Yes	0.73	0.38	0.87	0.01	5.31
DF3 (m)	No	9851	7954	9088	0	69682
	Yes	8285	6790	6926	0	41567
DT13 (m)	No	11737	7230	13279	0	92079
	Yes	8276	6231	8466	0	40780
DS (m)	No	6013	4563	5986	0	31673
	Yes	5477	4598	5310	0	30000
N (m)	No	1916917	1890920	103996	1599230	2094193
	Yes	1837517	1829803	152859	1528434	2096422
E (m)	No	1476552	1495012	95637	1277252	1749714
	Yes	1391353	1390049	51366	1297841	1569163
PRTA (%)	No	13.7	0.8	28.0	0	100
	Yes	23.4	1.5	36.3	0	100
PFRT (%)	No	25.6	8.3	35.2	0	100
	Yes	38.0	16.0	41.6	0	100

Descriptive statistics for covariates and introgression metrics at the 558 stream sites (330 outside and 228 inside the historical range of rainbow trout) in the dataset. Yellowstone cutthroat trout introgression (YCTI) was observed at 31 sites outside and 6 sites inside the historical range of rainbow trout.

[a]RTrange = historical range of rainbow trout

T = mean August stream temperature (1993–2011); S = stream reach slope; CFM = mean day of water year (starting 1 October) when 50% of annual flow had discharged (1977–2006); W95 = number of days in winter (1 December–28 February) when flows were in the highest 5% for the year; MAF = mean annual flow; DF3 = distance to nearest reach with mean annual flow $\geq$ 2.83 m^3/s (100 ft^3/s); DT13 = distance to nearest reach with a mean August stream temperature $\geq$ 13˚C; DS = shortest distance to a reach with mean annual flow $\geq$ 2.83 m^3/s, mean August stream temperature $\geq$ 13˚C, or known source of rainbow trout (e.g., headwater lake, naturalized population, or stocking in the last 10 years); N = UTM northing coordinate; E = UTM easting coordinate; YCTI = sites with evidence of Yellowstone cutthroat trout introgression; PRTA = percentage of rainbow trout alleles at a site; PFRT = percentage of fish with rainbow trout alleles at a site.

doi:10.1371/journal.pone.0163563.t001

were not (mean PRTA, 23.4 vs. 13.7%; mean PFRT, 38.0 vs 25.6%) but both areas showed a wide spectrum of introgression.

We used logistic regression to relate three levels of admixture—1%, 10%, and 20% rainbow trout alleles at a site—to biotic and abiotic covariates. The first two levels of admixture are thresholds used by state management agencies for designating cutthroat trout populations as core conservation populations (the highest priority for management) or conservation populations [15,44]. The third level was that below which populations were considered to constitute good phenotypic and ecological representatives of westslope cutthroat trout during evaluation of this taxon for listing under the U.S. Endangered Species Act [45], although this conclusion has been disputed [46]. We also developed logistic regression equations for these thresholds using PFRT at a site as a dependent variable to permit consideration of additional studies.

Environmental covariates that we considered for inclusion in the models were climatic, geomorphic, biological, and zoogeographic descriptors identified in earlier studies as correlated with levels of admixture or influential in distribution models for cutthroat trout and rainbow trout (Table B in S1 File). Variables related to flow were obtained from the Western U.S. streamflow metrics website (www.fs.fed.us/rm/boise/AWA E/projects/modeled_stream_flow_metrics.shtml) as ArcGIS table attributes that linked directly to the NHDPlus network and were derived from the Variable Infiltration Capacity model [47,48]. Mean August stream temperature scenarios were obtained from the NorWeST website (www.fs.fed.us/rm/boise/AWAE/projects/NorWeST.htm l; [49]) and reach slope values from Value Added Attributes associated with NHDPlus [38]. We assumed that rainbow trout fully occupied all potential habitats in their historical range to which they had access, and because of the intensity of historical stocking (see below), also were capable of achieving a similar distribution in locations to which they were introduced. The presence or abundance of native or introduced rainbow trout, however, was not consistently described across the study area, so we represented propagule pressure by this species by measuring proximity to known wild or stocked populations or to habitats favorable to rainbow trout. These variables included the distance to streams with high mean annual discharge (those habitats likely to serve as spawning habitat for rainbow trout but not cutthroat trout; see Table B in S1 File), the distance to reaches with mean August stream temperatures of at least 13°C (at which rainbow trout occupancy peaked in this region; [40]), or the shortest distance to an indigenous, naturalized, or subsidized source of rainbow trout. These distances were calculated using a customized algorithm implemented in a geographic information system based on a raster stream network with a spatial resolution of 30 m. Information on rainbow trout presence or stocking was obtained from agency databases or online reports (http://fishandgame.idaho.gov/public/fish/stocking/; http://fwp.mt.gov/fishing/mFish/). The historical range for rainbow trout was taken from the literature [4,50]. The incidence of Yellowstone cutthroat trout *O. c. bouvieri* introgression was noted in the studies used in the analysis. We included latitude and longitude as candidate variables because of their potential to represent regional climatic or ecosystem gradients that were not directly measured [51]. Other variables sometimes referenced in the cutthroat trout hybridization literature such as elevation, road density, precipitation, or wildfires were not considered because they were surrogates for the stream covariates that were used, not broadly available across the study area, or of uncertain effect on the location and degree of introgression. We were not able to characterize the distribution of local geological and anthropogenic barriers to fish migration, which introduced some error in our predictions about the current and future likelihood of introgression in parts of the network.

To avoid potential issues with multicollinearity, we examined pairwise correlations among all variables (Table C in S1 File) and removed center of flow mass (CFM) from consideration because it was highly correlated with the percentage of high flows that took place during winter (W95; r = -0.84) and did not contribute a significant effect to any models during exploratory analyses. We used the glmulti package [52] in R [53] to conduct the regression analyses, and ranked models based on AIC. Because we wished to make consistent predictions across thresholds throughout the analysis area for the introgression metric (PRTA) most often used in conservation assessments [44], we sought a consensus model across the three introgression thresholds. We chose the top-ranked model for the 10% and 20% thresholds, which was within 3 AIC points of the top-ranked model for the 1% threshold. We calculated a best model for each PFRT threshold at a site for comparison. Variables in the selected models had variance inflation factors < 3.5 and most had 95% confidence intervals that did not overlap zero. We avoided including interaction terms to maximize interpretability of the models and because the predictive accuracy of our models was good (see Results). Moreover, interactions were plausible for many variable combinations, thus a meaningful a priori set would have been unwieldy

based on the 15 possible pairwise combinations in the highest-ranked models, let alone the 45 possible combinations in the original models. We assessed predictive accuracy of the final models using area under the receiver operating curve (AUC) values, a measure of the performance of a model in assigning cases into dichotomous classes which ranges from 0.5 for random assignment to 1.0 for a perfect model [54]. We also calculated 2 x 2 classification tables that balanced error rates of omission and commission, and evaluated robustness of the model results using 10-fold cross-validation [55] calculated with the package DAAG [56].

The consensus model—with parameter estimates specific to the PRTA threshold—and the best model for each of the three PFRT thresholds were used to create prediction maps for the stream network (Table D in S1 File) that showed the probability of rainbow trout introgression exceeding 1%, 10%, or 20%. These predictions were subsequently classified as above or below the three hybridization levels using model-specific probability thresholds (0.35–0.54) that balanced errors of omission and commission observed in the data from the 501 sites with data on PRTA that were used to develop the models. These predictions indicated substantially higher introgression where rainbow trout were native, and that introgression increased with stream temperature (see Results). Thus, we developed classification maps for six cutthroat trout hybridization scenarios to represent: 1) current conditions, 2) introgression outside the historical range of rainbow trout equilibrating at levels seen inside that range (equilibrium), 3) moderate climate warming (+0.5°C stream temperatures) 4), equilibrium introgression plus moderate climate warming, 5) extreme climate warming (+1.0°C stream temperatures), and 6) equilibrium introgression plus extreme climate warming. The climate scenarios represent the amount of stream warming expected in the next 50–100 years based on recently estimated historical warming rates of ~0.1°C/decade [32] and the assumption that global warming will continue on the trajectory established over the past several decades [57]. For each scenario, the amount of habitat below the introgression threshold was summarized by stream length and volume. We acknowledge that some habitats are unlikely to be occupied by either westslope cutthroat trout or rainbow trout for reasons other than those we have considered e.g., the presence of migration barriers precluding occupancy or invasions by other nonnative species. Thus our predictions are of the potential change in introgression based on habitat and biotic characteristics, subject to re-evaluation at local scales.

Results

Variables in logistic regression models representing climate and the potential for modern and ancient propagule pressure were significantly associated with levels of introgression between westslope cutthroat trout and rainbow trout (Table 2 and Table E in S1 File). Moreover, relations between the probability of exceeding the given levels of introgression and the independent variables were largely as hypothesized (Table B in S1 File) and were consistent among models for both introgression metrics and across the three thresholds (Table 3). In the consensus model, which was also the top model for 10% and 20% PRTA, the probability of exceeding that level of introgression was significantly associated with warmer water temperatures, larger streams, proximity to warmer habitats and to recent sources of rainbow trout propagules (whether up- or downstream), presence within the historical range of rainbow trout, and locations further east. Translating this model into a series of response curves (Fig 2) highlighted the offset between curves representing the same temperature values inside and outside the historical range of rainbow trout, which may constitute a hybridization deficit that will eventually be overcome in the latter. It also demonstrated, however, that even within the historical range of rainbow trout, high levels of introgression were unlikely in the coldest streams or those more than a few kilometers from rainbow trout or rainbow trout habitat. For this model, AUC values

Table 2. Model selection results.

Introgression metric	Model	AIC
PRTA > 1%	T + RTrange + DS + E + MAF + DT13 + W95 + S	575.87
	T + RTrange + DS + E + MAF + DT13 + W95 + YCTI + S	576.23
	T + RTrange + DS + MAF + E + DT13 + YCTI + S	576.72
	T + RTrange + DS + E + MAF + W95 + S	576.88
PRTA > 10%	**T + RTrange + DS + DT13 + MAF + E**	433.13
	T + RTrange + DS + DT13 + MAF + E + YCTI	433.51
	T + RTrange + DS + DT13 + MAF + E + W95	434.27
	T + RTrange + DS + DT13 + MAF + E + S	434.32
PRTA > 20%	**T + RTrange + DT13 + MAF + E + DS**	387.75
	T + RTrange + DT13 + MAF + E	388.40
	T + RTrange + DT13 + MAF + E + DS + S	389.23
	T + RTrange + DT13 + MAF + E + DS + W95	389.35
PFRT > 1%	T + DS + DF3 + MAF + E + YCTI + RTrange + DT13	552.82
	T + DS + DF3 + MAF + E + YCTI + DT13	553.25
	T + DS + DF3 + MAF + E + YCTI + RTrange + DT13 + S	554.45
	T + DS + DF3 + MAF + E + YCTI + RTrange	554.72
PFRT > 10%	T + RTrange + DS + MAF + E + YCTI + S	551.44
	T + RTrange + DS + MAF + E + YCTI	552.05
	T + RTrange + DS + MAF + E + YCTI + S + DF3	553.14
	T + RTrange + DS + MAF + E + YCTI + S + W95	553.33
PFRT > 20%	T + RTrange + DS + MAF + E + YCTI + S	497.57
	T + RTrange + DS + MAF + E + YCTI	498.20
	T + RTrange + DS + MAF + E + YCTI + S + DF3	499.15
	T + RTrange + DS + MAF + E + YCTI + S + DT13	499.19

Model selection results for logistic regression equations relating environmental covariates to whether sites exceeded 1%, 10%, or 20% rainbow trout alleles (PRTA) or fish with rainbow trout alleles (PFRT). The four top models are ranked from most to least plausible for each metric and threshold. Underlined variables had coefficients that were not significantly different from zero. The model shown in bold font (the consensus model) was used to predict the probability that hybridization would exceed specified thresholds of PRTA in streams across the study area.

doi:10.1371/journal.pone.0163563.t002

were indicative of good model performance (0.78–0.86), classification success was high (72–82%), and 10-fold cross validation implied model results were robust (70–82%) across the three levels of introgression. The best models for PFRT at a site relied on a similar suite of covariates, with proximity to large habitats appearing in one model, a nonsignificant effect of slope appearing in two others, and a significant effect of the incidence of Yellowstone cutthroat trout introgression in all models (Table 3). Contrary to expectation, evidence of Yellowstone cutthroat trout hybridization was negatively related to exceeding thresholds in the percentage of fish with rainbow trout alleles. Performance of these models was also good (AUC, 0.80–0.84; classification success, 74–77%; cross-validation, 72–76%). For a common set of covariate values, exceeding thresholds was more likely for PFRT alleles than for PRTA at a site, but these metrics were highly correlated ($r = 0.89$). Model fit improved as the introgression threshold increased for both measures of introgression.

Application of logistic regression models to predict the probabilities of rainbow trout introgression exceeding 1%, 10%, or 20% throughout the stream network consistently showed that

Table 3. Model parameter estimates.

Introgression							Classification accuracy		
Metric	Predictor	b_x	SE	*z*	*p*	AUC	Threshold[a]	Training data	10-fold CV
PRTA > 1%	Intercept	-1.05E+01	2.20E+00	-4.77	<0.01	0.78	0.461	71.7%	69.4%
	T	2.91E-01	8.30E-02	3.51	<0.01				
	RTrange	7.73E-01	2.44E-01	3.16	<0.01				
	DS	-7.72E-05	2.63E-05	-2.94	<0.01				
	DT13	-1.90E-05	1.32E-05	-1.44	0.15				
	MAF	6.64E-01	1.95E-01	3.40	<0.01				
	E	4.94E-06	1.28E-06	3.87	<0.01				
PRTA > 10%	Intercept	-1.34E+01	2.65E+00	-5.07	<0.01	0.85	0.384	81.2%	81.0%
	T	2.66E-01	1.00E-01	2.65	<0.01				
	RTrange	1.34E+00	2.94E-01	4.57	<0.01				
	DS	-1.34E-04	4.24E-05	-3.16	<0.01				
	DT13	-6.18E-05	2.35E-05	-2.63	<0.01				
	MAF	5.12E-01	1.90E-01	2.69	<0.01				
	E	6.75E-06	1.51E-06	4.46	<0.01				
PRTA > 20%	Intercept	-1.33E+01	2.84E+00	-4.67	<0.01	0.86	0.353	82.2%	81.7%
	T	3.37E-01	1.09E-01	3.10	<0.01				
	RTrange	1.19E+00	3.09E-01	3.84	<0.01				
	DS	-8.11E-05	4.95E-05	-1.64	0.10				
	DT13	-7.97E-05	3.15E-05	-2.53	0.01				
	MAF	5.01E-01	1.93E-01	2.60	<0.01				
	E	5.74E-06	1.60E-06	3.58	<0.01				
PFRT >1%	Intercept	-9.29E+00	2.37E+00	-3.92	<0.01	0.80	0.532	74.4%	72.2%
	T	3.70E-01	9.11E-02	4.06	<0.01				
	RTrange	3.87E-01	2.48E-01	1.56	0.12				
	DS	-1.52E-04	3.37E-05	-4.52	<0.01				
	DF3	5.45E-05	1.93E-05	2.83	<0.01				
	MAF	1.01E+00	2.54E-01	3.96	<0.01				
	E	3.64E-06	1.40E-06	2.59	<0.01				
	YCTI	2.05E+00	5.50E-01	3.73	<0.01				
	DT13	2.56E-05	1.37E-05	1.88	0.06				
PFRT >10%	Intercept	-1.09E+01	2.66E+00	-4.11	<0.01	0.82	0.478	73.8%	73.0%
	T	4.28E-01	8.66E-02	4.94	<0.01				
	RTrange	5.24E-01	2.49E-01	2.10	0.04				
	DS	-1.04E-04	2.69E-05	-3.88	<0.01				
	MAF	5.69E-01	2.06E-01	2.77	<0.01				
	E	4.46E-06	1.44E-06	3.10	<0.01				
	YCTI	1.57E+00	4.54E-01	3.45	<0.01				
	S	-5.68E-02	3.53E-02	-1.61	0.11				
PFRT >20%	Intercept	-1.31E+01	2.85E+00	-4.60	<0.01	0.84	0.421	76.6%	76.1%
	T	5.17E-01	9.42E-02	5.49	<0.01				
	RTrange	7.37E-01	2.66E-01	2.77	<0.01				
	DS	-1.22E-04	3.27E-05	-3.72	<0.01				
	MAF	4.20E-01	1.89E-01	2.23	0.03				
	E	4.97E-06	1.52E-06	3.28	<0.01				
	YCTI	1.07E+00	4.66E-01	2.30	0.02				

(*Continued*)

Table 3. (*Continued*)

Introgression							Classification accuracy		
Metric	Predictor	b_x	SE	*z*	*p*	AUC	Threshold[a]	Training data	10-fold CV
	S	-6.17E-02	3.85E-02	-1.60	0.11				

Parameter estimates and summary statistics for the consensus model predicting the percentage of rainbow trout alleles at a site (PRTA) and the individual models for predicting the percentage of fish with rainbow trout alleles at a site (PFRT).

[a]Classification thresholds were based on values that yielded equal numbers of the classification errors "0 predicted but 1 observed" and "1 predicted but 0 observed" in the training datasets.

doi:10.1371/journal.pone.0163563.t003

probabilities were low in high mountain ranges with cold streams and higher in larger and warmer streams (Fig 3). Classification scenario maps indicated that locations with cutthroat trout populations exhibiting hybridization below 10% PRTA were potentially widespread (Fig 4A) and represented–31,622 km of habitat (Table 4). Assuming that levels of introgression outside the historical range of rainbow trout equilibrate at the level found where both species are native, we forecast that expanding hybrid zones will lead to 41.5–49.7% declines in the habitat occupied by nonintrogressed cutthroat trout populations (Fig 4B). Predicated on the notion that warming temperatures will favor the advance of rainbow trout alleles, changes in climate expected by mid-century were predicted to lead to modest reductions (9.5–26.5%) in the amount of habitat below each of the introgression thresholds (Fig 4C). Extreme changes in climate expected by late century led to further reductions (28.2–45.0% relative to current conditions) in habitat across these thresholds inside the historical range of rainbow trout (Fig 4E), and even greater declines (52.6–74.7%) outside that range if equilibrium conditions are also reached, yet not to the wholesale loss of nonintrogressed populations of cutthroat trout in many locations (Fig 4F). Projected declines were greater when summarized by volume than by length because larger streams at lower elevations were more likely to exceed the introgression thresholds as temperatures warmed (Table 4).

Discussion

Through the examination of hybrid zones replicated across much of the northern Rocky Mountains, we demonstrated that the geographic distribution of introgression between westslope cutthroat trout and rainbow trout can be accurately modeled by using variables representing ecosystem and zoogeographic properties. This runs counter to the notion that genomic extinction for cutthroat trout is inevitable following secondary contact with rainbow trout, but reaffirms that introgression between them is likely and predictable where their distributions overlap. As in many hybrid zones involving freshwater taxa [19,58–60], environmental gradients corresponded to clinal trends in allele frequencies. Although this correspondence is sometimes regarded as coincidental where hybrid zone location is driven by parental dispersal rates and hybrid individuals are less fit i.e., in tension zones [43], it is also regarded as evidence of isolation by environment between species with divergent ecological requirements [20]. Most of the variables in our top models are those which explain differences in habitat occupancy by the parental forms, supporting the notion of ecological segregation between these taxa [23,61]. Moreover, there is ample evidence of physiological differences between them that generates these differences. In general, hatchery and wild rainbow trout exhibit higher metabolic and oxygen consumption rates and lower food conversion efficiencies that equate to better performance in warmer, higher productivity environments ([21]; their Table 3), and in turn contribute to the likelihood of developing migratory life histories [62,63], of which anadromy is an

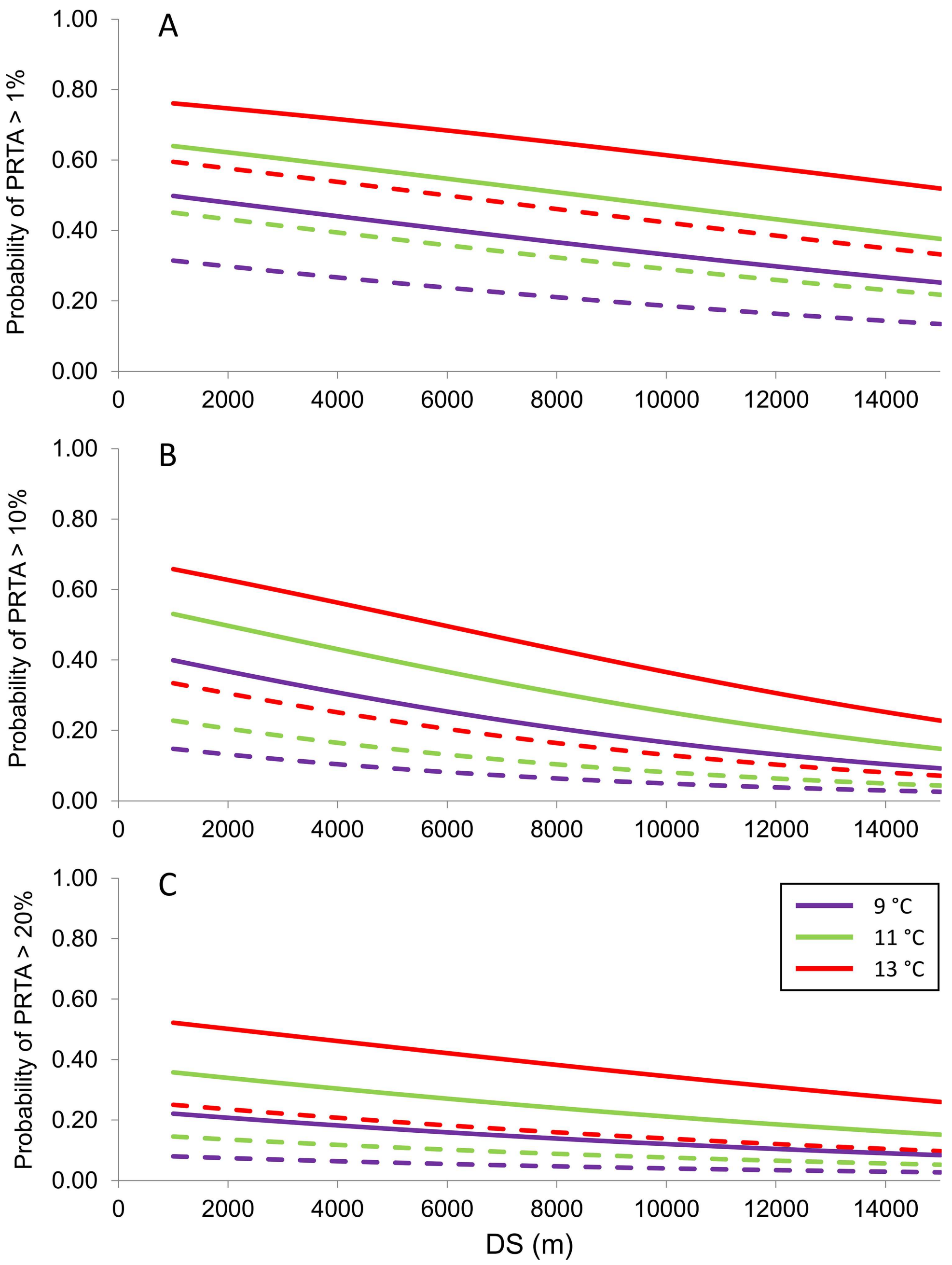

Fig 2. Covariate relations to introgression. Relations between the probability of exceeding the threshold level (1%, 10%, or 20%) of the percentage of rainbow trout alleles at a site (PRTA) and the distance to the nearest potential source of rainbow trout (DS) for three values of mean August stream temperature inside (solid lines) and outside (dashed lines) the historical range of rainbow trout. Other model covariates were set to their median values.

doi:10.1371/journal.pone.0163563.g002

extreme example. In contrast, westslope cutthroat trout (and perhaps Yellowstone cutthroat trout and coastal cutthroat trout *O. c. clarkii* [21,64,65]) have lower metabolic and oxygen consumption rates but higher food conversion efficiencies, traits that likely improve fitness in the colder, less productive, and smaller streams that characterize their present strongholds [15,40,66]. That no inland form of cutthroat trout exhibits anadromy, and that coastal

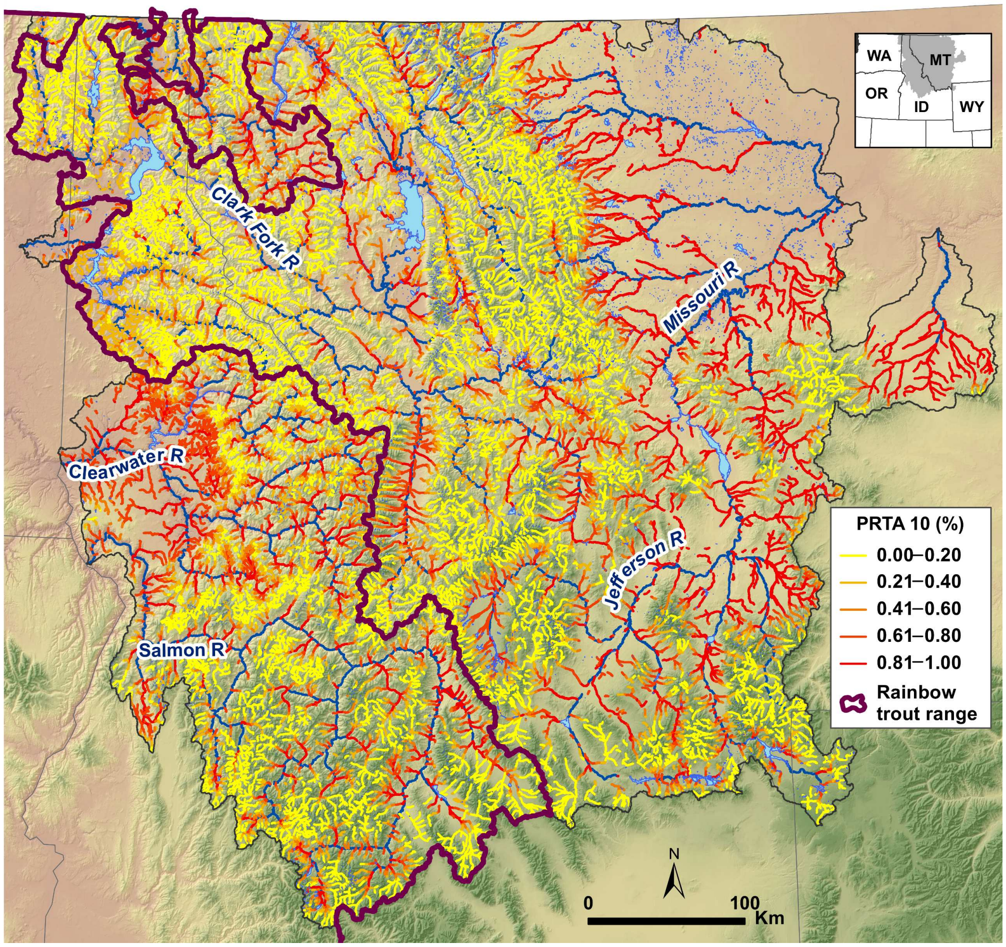

Fig 3. Predictions of 10% PRTA in the study area. Probabilities that the percentage of rainbow trout alleles at a site will exceed 10% (PRTA 10) across the range of westslope cutthroat trout in Montana and Idaho under current conditions. Blue river segments had mean annual flows > 7 m^3/s where model predictions were not made.

doi:10.1371/journal.pone.0163563.g003

cutthroat trout exhibit anadromy in a much reduced form [67], is also consistent with the species-level dichotomy. Additional comparisons of physiological traits among other subspecies of cutthroat trout and wild and naturalized stocks of rainbow trout would determine whether this difference is maintained across all members of each species. Regardless, this physiological divide appears to explain a number of aspects of the distribution and life histories of freshwater taxa, such as local or latitudinal trends in habitat occupancy [68,69] and sex-specific adoption of resident or anadromous life histories [70].

Although ecological segregation might be thought to limit introgressive hybridization, the development of complete pre- or post-zygotic reproductive barriers often lags millions of years behind genetic and presumably ecological divergence [71,72]. It has been argued that at the very least these reproductive barriers would be stronger for rainbow trout and cutthroat trout where both are native [73], presumably because of a longer period for reinforcement following secondary contact [74]. Yet we observed the opposite: levels of introgression for a given set of environmental characteristics were substantially higher where these species have co-occurred at least since the last glacial maximum than where rainbow trout have been introduced during the last two centuries. That such a difference exists is remarkable given the propagule pressure

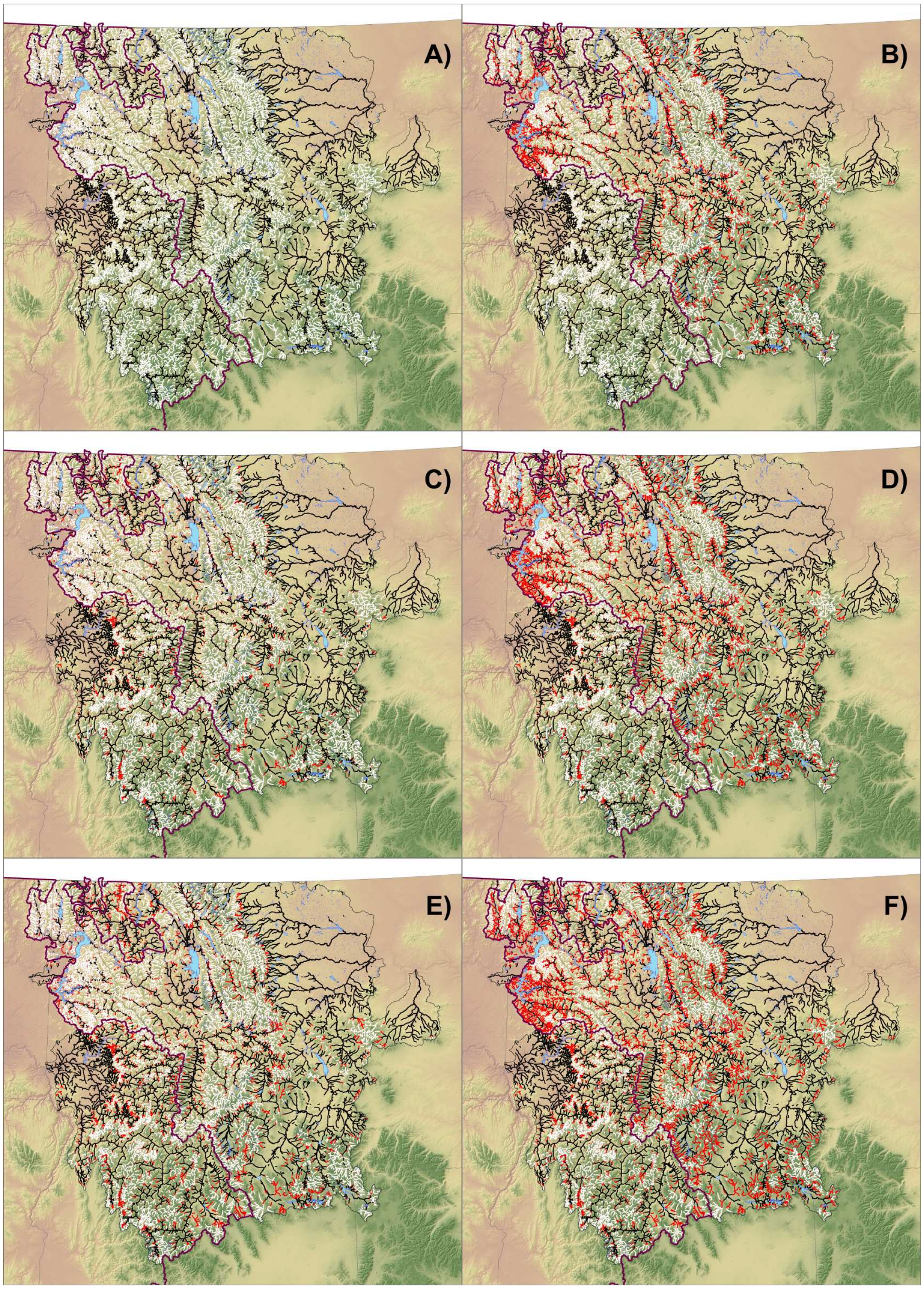

Fig 4. Predictions of 10% PRTA under multiple scenarios. Prediction maps showing whether stream reaches are expected to remain below the classification threshold (0.384) for 10% rainbow trout alleles at a site (stream reaches in black, no; in white, yes). Panel A shows the current scenario and remaining panels show predictions relative to current conditions. Scenarios match those in Table 4 for current (panel A), equilibrium (panel B), current +0.5˚C (panel C), equilibrium +0.5˚C (panel D), current +1.0˚C (panel E), and equilibrium +1.0˚C (panel F). Reaches in red are those expected to exceed this threshold relative to the current scenario.

doi:10.1371/journal.pone.0163563.g004

Table 4. Cutthroat trout habitat amounts relative to rainbow trout range.

		Inside range of rainbow trout				Outside range of rainbow trout			
Scenario[a]	PRTA	Length	% change	Volume	% change	Length	% change	Volume	% change
Current	<1%	7,869	—	1,656	—	16,186	—	4,117	—
	<10%	9,863	—	2,854	—	21,760	—	7,925	—
	<20%	10,887	—	3,394	—	23,290	—	9,051	—
Equilibrium	<1%	7,869	—	1,656	—	9,472	-41.5%	2,070	-49.7%
	<10%	9,863	—	2,854	—	13,643	-37.3%	4,479	-43.5%
	<20%	10,887	—	3,394	—	16,588	-28.8%	5,922	-34.6%
Current + 0.5˚C	<1%	6,302	-19.9%	1,245	-24.8%	13,855	-14.8%	116,017	-18.8%
	<10%	8,121	-17.7%	2,099	-26.5%	19,391	-10.2%	227,997	-16.0%
	<20%	9,339	-14.2%	2,582	-23.9%	20,882	-9.5%	264,298	-14.6%
Equilibrium + 0.5˚C	<1%	6,302	—	1,245	—	7,163	-55.7%	1,495	-63.7%
	<10%	8,121	—	2,099	—	11,110	-48.9%	3,402	-57.1%
	<20%	9,339	—	2,582	—	13,855	-40.5%	4,711	-47.9%
Current + 1.0˚C	<1%	4,962	-36.9%	911	-45.0%	11,240	-30.6%	2,620	-36.4%
	<10%	6,723	-31.8%	1,615	-43.4%	17,165	-21.1%	5,506	-30.5%
	<20%	7,820	-28.2%	1,982	-41.6%	18,746	-19.5%	6,438	-28.9%
Equilibrium + 1.0˚C	<1%	4,962	—	911	—	5,122	-68.4%	1,043	-74.7%
	<10%	6,723	—	1,615	—	8,455	-61.1%	2,475	-68.8%
	<20%	7,820	—	1,982	—	11,038	-52.6%	3,546	-60.8%

Length (km) and volume (m^3) of stream habitat inside and outside the historical range of rainbow trout predicted to host cutthroat trout populations with admixture below specified thresholds of the percentage of rainbow trout alleles (PRTA) at a site for the 55,234-km study area network. Estimates were obtained by applying the final predictive models to the covariates associated with each reach in the stream network.

[a]Geological barriers and non-native brook trout exclude cutthroat trout from some streams within the study area so estimates of habitat amount are optimistic, although barriers may preclude rainbow trout access in some streams. The estimates are also predicated on no changes in propagule pressure from rainbow trout, although this could be altered by stocking practices.

doi:10.1371/journal.pone.0163563.t004

associated with introductions of hatchery fish; in Montana alone, which is largely outside the historical range of rainbow trout, a conservative estimate is that 400 million have been introduced since 1924 (http://fwp.mt.gov/fishing/mFish/). That waters in Idaho were also heavily stocked—160 million rainbow trout were released in the Salmon River basin from 1967 to 2010 [29]—implies that propagule pressure from stocked fish seems unlikely to account for the geographical difference, hence we offer three alternative explanations. First, perhaps introduced stocks of rainbow trout have lower fitness than native populations, have been unable to spread as extensively, and have already reached their environmentally mediated distribution. A number of freshwater taxa have shown surprising resistance to introgression despite intense propagule pressure [75,76], a result which has often been attributed to lower fitness in the introduced forms. Such reduced fitness may also characterize hatchery-derived populations of salmonids [77], although it generally applies to captively reared fish, not naturalized populations [78]. Second, despite the widespread introduction of rainbow trout, there may be a number of locations to which it has yet to gain access and become established but is likely to do so i.e., two centuries has been insufficient for rainbow trout to fully invade areas to which they have been introduced. This implies that the development of hybrid zones is a long-term and ongoing process, and that the duration of secondary contact between species influences hybrid zone characteristics [79,80]. Large-scale non-native species invasions and native species replacements in aquatic systems, however, are often observed over much shorter time scales [81]. Third, it is plausible that hybrid zones in much of the native range of rainbow trout are influenced by the

presence of steelhead [4], an anadromous life history form that matures at a much larger size, is more fecund, and could enhance propagule pressure. Absence of this form outside the historical range of rainbow trout may contribute to reduced propagule pressure despite intensive stocking and account for the reduced prevalence of introgression. Although we modeled the worst-case scenario for westslope cutthroat trout populations i.e., that levels of introgression with rainbow trout outside their native range would rise to those seen inside that range, the first and third explanations suggest that equilibration is unlikely.

Except where habitats containing cutthroat trout are secured by migration barriers to rainbow trout, climate change is expected to shift the position of hybrid zones involving these species [82]. Warming waters are predicted to move hybrid zones upstream (or downstream from headwater lake outlets) and increase the proximity to warm habitats that serve as springboards for rainbow trout propagules. The colder areas in which cutthroat trout are favored, however, are warming relatively slowly, which combined with their generally higher gradients translates to slow climate velocities (~400 m/decade [32]). In some instances, cutthroat trout populations adjacent to very cold upstream areas may expand as temperatures become more suitable [40]. Nevertheless, the size and number of habitats that host populations of westslope cutthroat trout with the highest conservation values are expected to shrink. The ultimate extent of introgression, however, is contingent on a number of factors that could either exacerbate or mitigate these trends. Supplementation of rainbow trout in a basin, beyond the natural capacity of that system to produce them, might lead to artificially heightened levels of introgression that would ease once stocking was curtailed or access to the stream network was inhibited. Relaxation of introgression in native taxa has been observed following the cessation of stocking elsewhere e.g., between native Guadalupe bass *Micropterus treculii* and introduced smallmouth bass *M. dolomieu* [83] or native and introduced stocks of lake trout *Salvelinus namaycush* [84]. Likewise, suppression or removal of naturalized rainbow trout populations could reduce the likelihood of substantial introgression [85], as could epizootics to which rainbow trout are more susceptible (e.g., whirling disease, but see [86]). Similarly, because brook trout *S. fontinalis*, an introduced salmonid from eastern North America, are widely distributed in the northern Rocky Mountains and linked to declines in headwater populations of cutthroat trout [87], their control may enhance propagule pressure from parental forms of cutthroat trout, in effect reducing propagule pressure from rainbow trout. Enhancement or establishment of migratory life histories within genetically intact population of cutthroat trout could play a similar role, their higher fecundity delivering large numbers of propagules to sites well upstream of locations favored by rainbow trout [88,89]. Management of riparian zones to enhance the production of cutthroat trout or to cool water temperatures might result in a similar trend. Having such a broad portfolio of options should give hope to biologists seeking to reduce levels of introgression. Perhaps as important is that these results indicate that there are many environments in which managers need not intervene because habitats are unlikely to be favorable for introgression between these taxa now or in the future [32,40]. Because resources for conservation actions are limited, recognizing these habitats can contribute to more strategic and efficient conservation planning.

Finally, model performance improved as introgression thresholds increased from 1% to 20%, but this was anticipated. Errors for some of the genotyping methods used in our analysis can exceed 1% (cf. [18,90]), which could lead to incorrect assignment of sites above or below a threshold. Likewise, some populations of westslope cutthroat trout may have natural polymorphisms such that they have alleles otherwise diagnostic for rainbow trout [46], which can lead to overestimates of introgression. And because rainbow trout alleles are not randomly distributed among individuals in sites with admixture [18], the relatively small samples of fish from each site could yield imprecise estimates of introgression. Because 307 (61%) of the sites with

estimates of PRTA were within 1% of the lowest threshold (0–2% rainbow trout introgression), performance of the logistic regression models for the lowest threshold would be particularly sensitive to these issues. In contrast, very few of the introgression estimates were within 1% of the other thresholds (10%, 7 sites; 20%, 5 sites), leading to more robust model predictions. For all models, more complete data on those factors that influence parental abundance or the opportunity for introgression, such as the presence of migration barriers, the environmental factors associated with longitude, or the presence of brook trout [87], could improve model performance.

Conclusions

In summary, we conclude that hybridization between rainbow trout and westslope cutthroat trout is ongoing but that ecological segregation between these taxa arbitrates the extent and location of their hybrid zones. Consequently, the genomic extinction of westslope cutthroat trout from introductions of rainbow trout seems unlikely, even should the more extreme patterns of introgression inside the historical range of rainbow trout be realized elsewhere. Climate change is expected to further erode the number and size of habitats likely to host largely nonintrogressed populations of westslope cutthroat trout, in turn subjecting these populations to the greater risks of extirpation associated with declining abundance and increasing fragmentation [49,91]. Nevertheless, the widespread distribution of habitats resistant to incursions by rainbow trout, and the ability of cutthroat trout to occupy small and sometimes isolated habitats for many generations [40,92,93], suggests that genetically intact populations of westslope cutthroat trout are likely to remain in some areas through this century. Whether similar patterns obtain for other subspecies of cutthroat trout that hybridize with rainbow trout is unclear, although comparable patterns in the longitudinal zonation of introgression are evident [94–96]. Although introgressive hybridization between taxa can have many outcomes e.g., the formation of novel taxa or the partial introgression of adaptive genes [30,97], the potential for complete admixture [98] has been a focus of much of the conservation literature. In some circumstances, this process constitutes a legitimate hazard [99,100]. But because stream environments are often characterized by pronounced and complex environmental gradients, and habitat occupancy by freshwater ectotherms is predictably related to such gradients, the environmental mediation of hybridization is a credible alternative hypothesis that can form the basis for directing conservation efforts.

Supporting Information

S1 File. Tables A–E. Table A. Studies used in the synthetic analysis. Data from studies included in the synthetic analysis of introgression between westslope cutthroat trout and rainbow trout (RT). These include the published source, number of sample sites inside and outside the historical range of rainbow trout, number of genotyped fish, marker type, and the means (ranges) for each dependent variable. **Table B. Candidate variables.** Candidate variables considered in the logistic models, the rationale for their inclusion and expected effect, and supporting citations. **Table C. Covariate correlation matrix.** Correlations among environmental covariates and introgression estimates at the 558 stream sites in the dataset. **Table D. Model selection results for 10% PRTA.** Model selection results for logistic regression equations relating environmental covariates to whether sites exceeded 10% rainbow trout alleles (PRTA). The 20 top models are ranked from most to least plausible. Underlined variables had coefficients that were not significantly different from zero. The top-ranked model is the consensus model. **Table E. Study region statistics.** Descriptive statistics for model covariates throughout the 55,234-km stream network in the study area.
(DOCX)

Acknowledgments

Funding for this work was provided by Region 1 of the U.S. Forest Service. Institutional support was provided by the National Genomics Center for Wildlife and Fish Conservation, supported by the Rocky Mountain Research Station and U.S. Forest Service Washington Office.

Author Contributions

Conceptualization: MKY DJI KSM TMW DMB KLP DLH DEN MKS.

Data curation: MKY DJI TMW DMB MRC MPC DLH.

Formal analysis: MKY DJI.

Funding acquisition: MKY DJI KSM MRC MPC MKS.

Investigation: MKY DJI TMW DMB KLP KJC MRC MPC DLH.

Methodology: MKY DJI KSM DLH DEN MKS.

Project administration: MKY DJI.

Resources: MKY DJI TMW DMB KLP MRC MPC.

Software: MKY DJI DLH DEN.

Supervision: MKY DJI KSM MRC MPC MKS.

Validation: MKY DJI DLH.

Visualization: MKY DJI DLH.

Writing – original draft: MKY DJI.

Writing – review & editing: MKY DJI KSM TMW DMB KLP KJC MRC MPC DLH DEN MKS.

References

1. Lodge DM. Biological invasions: lessons for ecology. Trends Ecol Evol. 1993; 8: 133–137. doi: 10.1016/0169-5347(93)90025-K PMID: 21236129
2. Allendorf FW, Luikart GH, Aitken SN. Conservation and the genetics of populations. 2nd ed. New York: Wiley-Blackwell. 2013.
3. Allendorf FW, Leary RF. Conservation and distribution of genetic variation in a polytypic species, the cutthroat trout. Conserv Biol. 1988; 2: 170–184.
4. Behnke R. Trout and salmon of North America. New York: Chanticleer Press. 2002.
5. Crête-Lafrenière A, Weir LK, Bernatchez L. Framing the Salmonidae family phylogenetic portrait: a more complete picture from increased taxon sampling. PLoS ONE. 2012; 7: e46662. doi: 10.1371/journal.pone.0046662 PMID: 23071608
6. Hitt NP, Frissell CA, Muhlfeld CC, Allendorf FW. Spread of hybridization between native westslope cutthroat trout, *Oncorhynchus clarki lewisi*, and nonnative rainbow trout, *Oncorhynchus mykiss*. Can J Fish Aquat Sci. 2003; 60: 1440–1451.
7. Rubidge EM, Taylor EB. An analysis of spatial and environmental factors influencing hybridization between native westslope cutthroat trout *(Oncorhynchus clarkii lewisi*) and introduced rainbow trout (*O. mykiss*) in the upper Kootenay River drainage, British Columbia. Conserv Genet. 2005; 6: 369–384.
8. Boyer MC, Muhlfeld CC, Allendorf FW. Rainbow trout (*Oncorhynchus mykiss*) invasion and the spread of hybridization with native westslope cutthroat trout (*Oncorhynchus clarkii lewisi*). Can J Fish Aquat Sci. 2008; 65: 658–669.

9. Ostberg CO, Chase DM. Temporal genetic monitoring of hybridization between native westslope cutthroat trout and introduced rainbow trout in the Stehekin River, Washington. Northwest Sci. 2012; 86: 198–211.
10. Epifanio J, Philipp D. Simulating the extinction of parental lineages from introgressive hybridization: the effects of fitness, initial proportions of parental taxa, and mate choice. Rev Fish Biol Fish. 2001; 10: 339–354.
11. Muhlfeld CC, McMahon TE, Boyer MC, Gresswell RE. Local habitat, watershed, and biotic factors influencing the spread of hybridization between native westslope cutthroat trout and introduced rainbow trout. Trans Am Fish Soc. 2009; 138: 1036–1051.
12. Ferguson MM, Danzmann RG, Allendorf FW. Developmental success of hybrids between two taxa of salmonid fishes with moderate structural gene divergence. Can J Zool. 1988; 66: 1389–1395.
13. Ostberg CO, Duda JJ, Graham JH, Zhang S, Haywood KP III, Miller B, et al. Growth, morphology, and developmental instability of rainbow trout, Yellowstone cutthroat trout, and four hybrid generations. Trans Am Fish Soc. 2011; 140: 334–344.
14. Kovach RP, Muhlfeld CC, Boyer MC, Lowe WH, Allendorf FW, Luikart G. Dispersal and selection mediate hybridization between a native and invasive species. Proc R Soc B. 2015; 282: 2014–2454.
15. Shepard BB, May BE, Urie W. Status and conservation of westslope cutthroat trout within the western United States. N Am J Fish Manag. 2005; 25: 1426–1440.
16. Williams I, Reeves GH, Graziano SL, Nielsen JL. Genetic investigation of natural hybridization between rainbow and coastal cutthroat trout in the Copper River Delta, Alaska. Trans Am Fish Soc. 2007; 136: 926–942.
17. Bingham DM, Buckskin P, Osborne H. Isolation, migration, and local recruitment drive persistence of cutthroat trout in tributaries near American Falls Reservoir. N Am J Fish Manag. 2016; 36: 590–605.
18. McKelvey KS, Young MK, Wilcox TM, Bingham DM, Pilgrim KL, Schwartz MK. Patterns of hybridization among cutthroat trout and rainbow trout in northern Rocky Mountain streams. Ecol Evol. 2016; 6: 688–706. doi: 10.1002/ece3.1887 PMID: 26865958
19. Culumber ZW, Fisher HS, Tobler M, Mateos M, Barber PH, Sorenson MD, et al. Replicated hybrid zones of *Xiphophorus* swordtails along an elevational gradient. Mol Ecol. 2011; 20: 342–356. doi: 10.1111/j.1365-294X.2010.04949.x PMID: 21210879
20. Culumber ZW, Shepard DB, Coleman SW, Rosenthal GG, Tobler M. Physiological adaptation along environmental gradients and replicated hybrid zone structure in swordtails (Teleostei: *Xiphophorus*). J Evol Biol. 2012; 25: 1800–1814. doi: 10.1111/j.1420-9101.2012.02562.x PMID: 22827312
21. Rasmussen JB, Robinson MD, Hontela A, Heath DD. Metabolic traits of westslope cutthroat trout, introduced rainbow trout and their hybrids in an ecotonal hybrid zone along an elevation gradient. Biol J Linn Soc Lond. 2012; 105: 56–72.
22. Wang IJ, Bradburd GS. Isolation by environment. Mol Ecol. 2014; 23: 5649–5662. doi: 10.1111/mec.12938 PMID: 25256562
23. Wenger SJ, Isaak DJ, Dunham JB, Fausch KD, Luce CH, Neville HM, et al. Role of climate and invasive species in structuring trout distributions in the interior Columbia River Basin, USA. Can J Fish Aquat Sci. 2011; 68: 988–1008.
24. Corse E, Pech N, Sinama M, Costedoat C, Chappaz R, Gilles A. When anthropogenic river disturbance decreases hybridisation between non-native and endemic cyprinids and drives an ecomorphological displacement towards juvenile state in both species. PLoS ONE. 2015; 10: e0142592. doi: 10.1371/journal.pone.0142592 PMID: 26561027
25. Splendiani A, Ruggeri P, Giovannotti M, Pesaresi S, Occhipinti G, Fioravanti T, et al. Alien brown trout invasion of the Italian peninsula: the role of geological, climate and anthropogenic factors. Biol Inv. 2016; 1–16. doi: 10.1007/s10530-016-1149-7
26. Lockwood JL, Cassey P, Blackburn T. The role of propagule pressure in explaining species invasions. Trends Ecol Evol. 2005; 31: 223–228.
27. Rahel FJ. Homogenization of freshwater faunas. Annu Rev Ecol Syst. 2002; 33: 291–315.
28. Dunham JB, Pilliod DS, Young MK. Assessing the consequences of nonnative trout in headwater ecosystems in western North America. Fisheries. 2004; 29(6): 18–26.
29. Loxterman JL, Keeley ER, Njoroge ZM. Evaluating the influence of stocking history and barriers to movement on the spatial extent of hybridization between westslope cutthroat trout and rainbow trout. Can J Fish Aquat Sci. 2014; 71: 1050–1058.
30. Abbott R, Albach D, Ansell S, Arntzen JW, Baird SJE, Bierne N, et al. Hybridization and speciation. J Evol Biol. 2013; 26: 229–246. doi: 10.1111/j.1420-9101.2012.02599.x PMID: 23323997

31. Isaak DJ, Rieman BE. Stream isotherm shifts from climate change and implications for distributions of ectothermic organisms. Glob Chang Biol. 2013; 19: 742–751. doi: 10.1111/gcb.12073 PMID: 23504832

32. Isaak D, Young M, Luce C, Hostetler S, Wenger S, Peterson E, et al. Slow climate velocities of mountain streams portend their role as refugia for cold-water biodiversity. Proc Nat Acad Sci. 2016; 113: 4374–4379. doi: 10.1073/pnas.1522429113 PMID: 27044091

33. Taylor SA, Larson EL, Harrison RG. Hybrid zones: windows on climate change. Trends Ecol Evol. 2015; 30: 398–406. doi: 10.1016/j.tree.2015.04.010 PMID: 25982153

34. Weigel DE, Peterson JT, Spruell P. Introgressive hybridization between native cutthroat trout and introduced rainbow trout. Ecol Appl. 2003; 13: 38–50.

35. Bennett SN, Olson JR, Kershner JL, Corbett P. Propagule pressure and stream characteristics influence introgression: cutthroat and rainbow trout in British Columbia. Ecol Appl. 2010; 20: 263–277. PMID: 20349846

36. Yau MM, Taylor EB. Environmental and anthropogenic correlates of hybridization between westslope cutthroat trout (*Oncorhynchus clarkii lewisi*) and introduced rainbow trout (*O. mykiss*). Conserv Genet. 2013; 14: 885–900.

37. Cooter W, Rineer J, Bergenroth B. A nationally consistent NHD-Plus framework for identifying interstate waters: implications for integrated assessments and interjurisdictional TMDLs. Environ Manag. 2010; 46: 510–524.

38. McKay L, Bondelid T, Dewald T, Johnston J, Moore R, Reah A. NHDPlus Version 2: User Guide. 2012. ftp://ftp.horizon-systems.com/NHDPlus/NHDPlusV21/Documentation/NHDPlusV2_User_Guide.pdf.

39. Poisot T, Gravel D, Leroux S, Wood SA, Fortin MJ, Baiser B, et al. Synthetic datasets and community tools for the rapid testing of ecological hypotheses. Ecography. 2015; 39: 402–408.

40. Isaak DJ, Young MK, Nagel DE, Horan DL, Groce MC. The cold-water climate shield: delineating refugia for preserving salmonid fishes through the 21st century. Glob Chang Biol. 2015; 21: 2540–2553.

41. Dunnigan JL. The spatial distribution of cutthroat trout in the Coeur d' Alene River system, Idaho. Master's thesis, University of Idaho, Moscow. 1997. 65 p.

42. Rieman BE, Dunham JB. Metapopulations and salmonids: a synthesis of life history patterns and empirical observations. Ecol Freshw Fish. 2000; 9: 51–64.

43. Barton NH, Hewitt GM. Analysis of hybrid zones. Annu Rev Ecol Syst. 1985; 16: 113–148.

44. Utah Division of Wildlife Resources. Cutthroat trout management: a position paper. Genetic considerations associated with cutthroat trout management. Utah Division of Publication 00–26, Utah Division of Wildlife Resources, Salt Lake City. 9 p. 2000. Available: https://cpw.state.co.us/Documents/Research/Aquatic/CutthroatTrout/CutthroatGeneticConsiderations29Dec2000.pdf.

45. U.S. Fish and Wildlife Service. Endangered and threatened wildlife and plants: reconsidered finding for an amended petition to list the westslope cutthroat trout as threatened throughout its range. Fed Regist. 2003; 68: 46989–47009.

46. Allendorf FW, Leary RF, Hitt NP, Knudsen KL, Lundquist LL, Spruell P. Intercrosses and the U.S. Endangered Species Act: should hybridized populations be included as westslope cutthroat trout? Conserv Biol. 2004; 18: 1203–1213.

47. Elsner MM, Cuo L, Voisin N, Deems JS, Hamlet AF, Vano JA, et al. Implications of 21st century climate change for the hydrology of Washington state. Clim Chang. 2010; 102: 225–260.

48. Wenger SJ, Luce CH, Hamlet AF, Isaak DJ, Neville HM. Macroscale hydrologic modeling of ecologically relevant flow metrics. Wat Resour Res. 2010; 46: W09513.

49. Isaak DJ, Luce CH, Rieman BE, Nagel DE, Peterson EE, Horan DL, et al. Effects of climate change and wildfire on stream temperatures and salmonid thermal habitat in a mountain river network. Ecol Appl. 2010; 20: 1350–1371. PMID: 20666254

50. Muhlfeld CC, Albeke SE, Gunckel SL, Writer BJ, Shepard BB, May BE. Status and conservation of interior Redband Trout in the western United States. N Am J Fish Manag. 2015; 25: 31–53.

51. Beale CM, Lennon JJ, Yearsley JM, Brewer MJ, Elston DA. Regression analysis of spatial data. Ecol Lett. 2010; 13: 246–264. doi: 10.1111/j.1461-0248.2009.01422.x PMID: 20102373

52. Calcagno V. glmulti: model selection and multimodel inference made easy. R package version 1.0.7. https://CRAN.R-project.org/package=glmulti. 2013. Accessed 15 May 2016.

53. R Core Team. R: A language and environment for statistical computing. R Foundation for Statistical Computing, Vienna, Austria. 2015. Available: https://www.R-project.org/.

54. Hosmer DW, Lemeshow S. Applied logistic regression. New York: John Wiley & Sons. 2000.

55. Boyce MS, Vernier PR, Nielsen SE, Schmiegelow FKA. Evaluating resource selection functions. Ecol Modelling. 2002; 157: 281–300.

56. Maindonald J, Braun WJ. DAAG: Data Analysis and Graphics Data and Functions. R package version 1.22. 2015. Available: https://CRAN.R-project.org/package=DAAG. Accessed 15 May 2016.

57. Foster G, Rahmstorf S. Global temperature evolution 1979–2010. Environ Res Lett. 2011; 6, 044022. doi: 10.1088/1748-9326/6/4/044022

58. Vonlanthen P, Roy D, Hudson AG, Largiader CR, Bittner D, Seehausen O. Divergence along a steep ecological gradient in lake whitefish (*Coregonus* sp.). J Evol Biol. 2009; 22: 498–514. doi: 10.1111/j.1420-9101.2008.01670.x PMID: 19170819

59. Duvernell DD, Schaefer JF. Variation in contact zone dynamics between two species of topminnows, *Fundulus notatus* and *F. olivaceus*, across isolated drainage systems. Evol Ecol. 2014; 28: 37–53.

60. Stoffels RJ, Richardson AJ, Vogel MT, Coates SP, Müller WJ. What do metabolic rates tell us about thermal niches? Mechanisms driving crayfish distributions along an altitudinal gradient. Oecologia. 2016; 180: 45–54. doi: 10.1007/s00442-015-3463-7 PMID: 26440800

61. Wenger SJ, Isaak DJ, Luce CH, Neville HM, Fausch KD, Dunham JB, et al. Flow regime, temperature, and biotic interactions drive differential declines of trout species under climate change. Proc Nat Acad Sci. 2011b; 108: 14175–14180.

62. Corsi MP, Eby LA, Barfoot CA. Hybridization with rainbow trout alters life history traits of native westslope cutthroat trout. Can J Fish Aquat Sci. 2013; 70: 895–904.

63. Kendall NW, McMillan JR, Sloat MR, Buehrens TW, Quinn TP, Pess GR, et al. Anadromy and residency in steelhead and rainbow trout (*Oncorhynchus mykiss*): a review of the processes and patterns. Can J Fish Aquat Sci. 2014; 72: 319–342.

64. Dwyer WP, Kramer RH. Influence of temperature on scope for activity in cutthroat trout, *Salmo clarki*. Trans Am Fish Soc. 1975; 104: 552–554.

65. Kindschi GA, Koby RF. Performance and oxygen consumption of Snake River cutthroat trout reared at four densities with supplemental oxygen. Prog Fish Cult. 1994; 56: 13–18.

66. Gresswell RE. Biology, status, and management of the Yellowstone cutthroat trout. N Am J Fish Manag. 2011; 31:782–812.

67. Quinn TP, Myers KW. Anadromy and the marine migrations of Pacific salmon and trout: Rounsefell revisited. Rev Fish Biol Fish. 2004; 14: 421–442.

68. Finstad AG, Forseth T, Jonsson B, Bellier E, Hesthagen T, Jensen AJ, et al. Competitive exclusion along climate gradients: energy efficiency influences the distribution of two salmonid fishes. Glob Chang Biol. 2011; 17: 1703–1711.

69. Van Leeuwen TE, Rosenfeld JS, Richards JG. Adaptive trade-offs in juvenile salmonid metabolism associated with habitat partitioning between coho salmon and steelhead trout in coastal streams. J Anim Ecol. 2011; 80: 1012–1023. doi: 10.1111/j.1365-2656.2011.01841.x PMID: 21466553

70. Sloat MR, Reeves GH. Individual condition, standard metabolic rate, and rearing temperature influence steelhead and rainbow trout (*Oncorhynchus mykiss*) life histories. Can J Fish Aquat Sci. 2014; 71: 491–501.

71. Aboim MA, Mavarez J, Bernatchez L, Coelho MM. Introgressive hybridization between two Iberian endemic cyprinid fish: a comparison between two independent hybrid zones. J Evol Biol. 2010; 23: 817–828. doi: 10.1111/j.1420-9101.2010.01953.x PMID: 20210830

72. Bossu CM, Near TJ. Characterization of a contemporaneous hybrid zone between two darter species (*Etheostoma bison* and *E. caeruleum*) in the Buffalo River system. Genetica. 2013; 141: 75–88. doi: 10.1007/s10709-013-9707-8 PMID: 23443763

73. Taylor EB. Evolution in mixed company: evolutionary inferences from studies of natural hybridization in Salmonidae. Hendry AP, Stearns SC, editors. Evolution illuminated: salmon and their relatives. Oxford University Press; 2004. pp. 232–263.

74. Servedio MR. The what and why of research on reinforcement. PLoS Biol. 2004; 2: e420. doi: 10.1371/journal.pbio.0020420 PMID: 15597115

75. Winkler KA, Pamminger-Lahnsteiner BA, Wanzenböck J, Weiss S. Hybridization and restricted gene flow between native and introduced stocks of alpine whitefish (*Coregonus* sp.) across multiple environments. Mol Ecol. 2011; 20: 456–472. doi: 10.1111/j.1365-294X.2010.04961.x PMID: 21199024

76. Perrier C, Guyomard R, Bagliniere JL, Nikolic N, Evanno G. Changes in the genetic structure of Atlantic salmon populations over four decades reveal substantial impacts of stocking and potential resiliency. Ecol Evol. 2013; 3: 2334–2349. doi: 10.1002/ece3.629 PMID: 23919174

77. Araki H, Berejikian BA, Ford MJ, Blouin MS. Fitness of hatchery-reared salmonids in the wild. Evol Appl. 2008; 1: 342–355. doi: 10.1111/j.1752-4571.2008.00026.x PMID: 25567636

78. Miller LM, Close T, Kapuscinski AR. Lower fitness of hatchery and hybrid rainbow trout compared to naturalized populations in Lake Superior tributaries. Mol Ecol. 2004; 13: 3379–3388. doi: 10.1111/j.1365-294X.2004.02347.x PMID: 15487997

79. Curry CM, Patten MA. Current and historical extent of phenotypic variation in the Tufted and Black-crested Titmouse (Paridae) hybrid zone in the southern Great Plains. Am Midl Nat. 2014; 171: 271–300.

80. Curry CM, Patten MA. Shadow of a doubt: premating and postmating isolating barriers in a temporally complex songbird (Passeriformes: Paridae) hybrid zone. Behav Ecol Sociobiol. 2016; 1–6.

81. Moyle PB, Light T. Biological invasions of fresh water: empirical rules and assembly theory. Biol Conserv. 1996; 78: 149–161.

82. Muhlfeld CC, Kovach RP, Jones LA, Al-Chokhachy R, Boyer MC, Leary RF, et al. Invasive hybridization in a threatened species is accelerated by climate change. Nat Clim Chang. 2014; 4: 620–624.

83. Bean PT, Lutz-Carrillo DJ, Bonner TH. Rangewide survey of the introgressive status of Guadalupe Bass: implications for conservation and management. Trans Am Fish Soc. 2013; 142: 681–689.

84. Valiquette E, Perrier C, Thibault I, Bernatchez L. Loss of genetic integrity in wild lake trout populations following stocking: insights from an exhaustive study of 72 lakes from Quebec, Canada. Evol Appl. 2014; 7: 625–644. doi: 10.1111/eva.12160 PMID: 25067947

85. Al-Chokhachy R, Muhlfeld CC, Boyer MC, Jones LA, Steed A, Kershner JL. Quantifying the effectiveness of conservation measures to control the spread of anthropogenic hybridization in stream salmonids: a climate adaptation case study. N Am J Fish Manag. 2014; 34: 642–652.

86. Carim K, Eby L, Pierce R. Does whirling disease mediate hybridization between a native and nonnative trout? N Am J Fish Manag. 2015; 35: 337–351.

87. Fausch KD, Rieman BE, Dunham JB, Young MK, Peterson DP. Invasion versus isolation: trade-offs in managing native salmonids with barriers to upstream movement. Conserv Biol. 2009; 23: 859–870. doi: 10.1111/j.1523-1739.2008.01159.x PMID: 19210302

88. Henderson R, Kershner JL, Toline CA. Timing and location of spawning by nonnative wild rainbow trout and native cutthroat trout in the South Fork Snake River, Idaho, with implications for hybridization. N Am J Fish Manag. 2000; 20: 584–596.

89. DeRito JN, Zale AV, Shepard BB. Temporal reproductive separation of fluvial Yellowstone cutthroat trout from rainbow trout and hybrids in the Yellowstone River. N Am J Fish Manag. 2010; 30: 866–886.

90. DeHaan PW, Schwabe LT, Ardren WR. Spatial patterns of hybridization between bull trout, *Salvelinus confluentus*, and brook trout, *Salvelinus fontinalis* in an Oregon stream network. Conserv Genet. 2010; 11: 935–949.

91. Haak AL, Williams JE. Spreading the risk: native trout management in a warmer and less-certain future. N Am J Fish Manag. 2012; 32: 387–401.

92. Young MK. Generation-scale movement patterns of cutthroat trout (*Oncorhynchus clarkii pleuriticus*) in a stream network. Can J Fish Aquat Sci. 2011; 68: 941–951.

93. Peterson DP, Rieman BE, Horan DL, Young MK. Patch size but not short-term isolation influences occurrence of westslope cutthroat trout above human-made barriers. Ecol Freshw Fish. 2014; 23: 556–571.

94. Ostberg CO, Slatton SL, Rodriguez RJ. Spatial partitioning and asymmetric hybridization among sympatric coastal steelhead trout (*Oncorhynchus mykiss irideus*), coastal cutthroat trout (*O. clarki clarki*) and interspecific hybrids. Mol Ecol. 2004; 13: 2773–2788. doi: 10.1111/j.1365-294X.2004.02268.x PMID: 15315688

95. Gunnell K, Tada MK, Hawthorne FA, Keeley ER, Ptacek MB. Geographic patterns of introgressive hybridization between native Yellowstone cutthroat trout (*Oncorhynchus clarkii bouvieri*) and introduced rainbow trout (*O. mykiss*) in the South Fork of the Snake River watershed, Idaho. Conserv Genet. 2008; 9: 49–64.

96. Buehrens TW, Glasgow J, Ostberg CO, Quinn TP. Spatial segregation of spawning habitat limits hybridization between sympatric native steelhead and coastal cutthroat trout. Trans Am Fish Soc. 2013; 142: 221–233.

97. Pereira RJ, Martínez-Solano I, Buckley D. Hybridization during altitudinal range shifts: nuclear introgression leads to extensive cyto-nuclear discordance in the fire salamander. Mol Ecol. 2016; doi: 10.1111/mec.13575 PMID: 26850834

98. Rhymer JM, Simberloff D. Extinction by hybridization and introgression. Annu Rev Ecol Syst. 1996; 27: 83–109.

99. Childs MR, Echelle AA, Dowling TE. Development of the hybrid swarm between Pecos pupfish (Cyprinodontidae: *Cyprinodon pecosensis*) and sheepshead minnow (*Cyprinodon variegatus*): a perspective from allozymes and mtDNA. Evolution. 1996; 50: 2014–2022.

100. Meraner A, Venturi A, Ficetola GF, Rossi S, Candiotto A, Gandolfi A. Massive invasion of exotic *Barbus barbus* and introgressive hybridization with endemic *Barbus plebejus* in northern Italy: where, how, and why? Mol Ecol. 2013; 22: 5295–5312. doi: 10.1111/mec.12470 PMID: 24103005
